# Site‐Specific Disulfide‐Mediated Crosslinking of DNA Nanocubes for Enhanced Biological Applications

**DOI:** 10.1002/smsc.202400471

**Published:** 2024-12-12

**Authors:** Sinan Faiad, Quentin Laurent, Jathavan Asohan, Tyler Brown, Alexander Prinzen, Hanadi Farouk Sleiman

**Affiliations:** ^1^ Department of Chemistry McGill University 801 Sherbrooke St West Montreal Québec H3A 0B8 Canada

**Keywords:** cellular uptake, crosslinking, disulfides, DNA nanotechnology, DNA nanocubes, dynamic covalent chemistry, physiological stability

## Abstract

The development of DNA nanotechnology has enabled the creation of diverse nanomaterials with significant potential in biological applications, such as sensing or drug delivery. From DNA origami to wireframe nanostructures, several strategies have been developed to deliver nucleic acid therapeutics into cells. However, these self‐assembled structures suffer from poor stability in biological media due to low concentrations of divalent cations, degradation by nucleases, and thermal denaturation. Herein, a site‐specific crosslinking method based on thiol‐disulfide exchange to stabilize a wireframe DNA nanocube is developed. With nearly quantitative crosslinking yields, the structure retains its structural integrity in conditions that mimic physiological environments. This results in improved cellular uptake, likely due to more favorable interaction with cell‐surface scavenger receptors, followed by endocytosis. This study paves the way for in vivo applications of DNA wireframe nanostructures by removing one of the major bottlenecks for their translation from in vitro to preclinical work.

## Graphical abstract

DNA nanocubes modified with site‐specific disulfide monomers are used to covalently crosslink the structure. Crosslinked nanocubes exhibit enhanced stability in serum due to improved resistance against exonucleases. This enhanced stability allows for the structural maintenance of the nanocube which facilitates cellular uptake via scavenger receptors. Non‐crosslinked nanocubes are degraded and do not engage the same uptake pathway into cells.

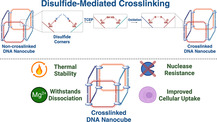

## 1 Introduction

Outside its role as the carrier of genetic information, DNA has emerged as a versatile nanomaterial with numerous applications ranging from sensing and imaging^[^ ^1^, ^2^ ^]^ to therapeutics and drug delivery.^[^ ^3^, ^4^ ^]^ Its programmable base pairing and straightforward synthesis have established this molecule as a key structural component for assembling nanostructures with precise control over shape and dimension. In addition, DNA's innate biocompatibility has facilitated its integration into various bioapplications. These advantages have naturally propelled DNA nanostructures toward functional in vivo use.

One class of DNA nanostructures, known as DNA origami, has been used for bioapplications such as stimuli‐responsive delivery of small molecule drugs,^[^ ^5^, ^6^ ^]^ biosensing of viruses,^[^ ^7^ ^]^ and the biocatalysis of enzymatic cascades.^[^ ^8^ ^]^ These nanostructures are typically constructed from hundreds of “staple” sequences that hybridize along a long circular DNA scaffold, folding it into a pre‐designed structure. A second class of nanostructure, known as DNA‐minimal wireframe structure, requires only a few DNA strands to self‐assemble into three‐dimensional forms. Notable examples of this are the DNA tetrahedron with strong potential for use in biosensing,^[^ ^9^, ^10^ ^]^ tissue engineering,^[^ ^11^ ^]^ and drug delivery,^[^ ^12^, ^13^ ^]^ and DNA prismatic structures that have demonstrated conditional delivery of siRNA in response to cancer biomarkers,^[^ ^14^ ^]^ ability to act as strong albumin binders,^[^ ^15^ ^]^ nanopores,^[^ ^16^ ^]^ templates for organizing nanomaterials,^[^ ^17^ ^]^ and for nanoprinting on polymers.^[^ ^18^ ^]^ The limited number of components involved in these systems make these types of nanostructures promising for potential clinical applications.

However, both DNA origami and wireframe nanostructures face significant challenges in physiological environments. This is primarily due to the presence of endogenous nucleases which rapidly degrade nucleic acids in the body.^[^ ^19^ ^]^ Moreover, the assembly of these structures often relies on high concentrations of divalent cations, which are not available physiologically at the necessary levels to maintain structural integrity.^[^ ^20^ ^]^

To address these issues, several strategies have been developed to stabilize DNA nanostructures. One approach involves protectively coating nanostructures with moieties such as serum albumin,^[^ ^21^ ^]^ polycationic peptides,^[^ ^22^ ^]^ silica,^[^ ^23^ ^]^ or polyethylene glycol polymers.^[^ ^24^ ^]^ While this has been shown to reduce nuclease‐mediated degradation, these external modifications can compromise functionality.^[^ ^25^ ^]^ Alternatively, covalent crosslinking has been used to stabilize nanostructures. These approaches typically involve using UV light,^[^ ^26^, ^27^, ^28^ ^]^ small molecules, or click chemistry^[^ ^29^, ^30^ ^]^ to covalently ligate supramolecular DNA assemblies. However, these crosslinking methods are usually irreversible, can have low selectivity, may impart unintended damage to DNA strands,^[^ ^31^ ^]^ and typically lead to heterogeneous final products due to incomplete crosslinking.^[^ ^26^ ^]^

Reversible disulfide crosslinking has been used on nanoparticles such as micelles,^[^ ^32^, ^33^, ^34^ ^]^ hydrogels,^[^ ^35^ ^]^ DNA tiles,^[^ ^36^ ^]^ and origami^[^ ^37^ ^]^ to temporarily increase stability while allowing for stimuli‐responsive disassembly in reducing conditions. This is especially convenient for the selective release of a therapeutic cargo only upon entry into the reductive cellular environment. In recent work from our lab, we demonstrated the controlled incorporation of disulfide moieties into DNA using phosphoramidite chemistry. This method allows for the precise introduction of abiological monomers at any position of a DNA sequence.^[^ ^38^, ^39^ ^]^ This led to the development of a DNA amphiphile that self‐assembles into a spherical nucleic acid (SNA) with a hydrophobic disulfide core, which can be covalently crosslinked.^[^ ^39^ ^]^

In this work, we design a DNA nanocube with strategically positioned disulfide units that can be employed to achieve near‐quantitative crosslinking yields. We elucidate the mechanism of crosslinking and demonstrate that the crosslinked nanostructure withstands dissociation in harsh conditions lacking divalent cations, resists denaturation at high temparatures, and does not dissociate in the presence of serum proteins. We study the degradation of the crosslinked nanocube in serum, demonstrating improved resistance to exonucleases. This stability translates to enhanced cellular penetration for the crosslinked nanocube compared to its non‐crosslinked counterpart.

## 2 Results and Discussion

### 2.1 Design and Crosslinking of Disulfide DNA Nanocube

**Figure 1:**
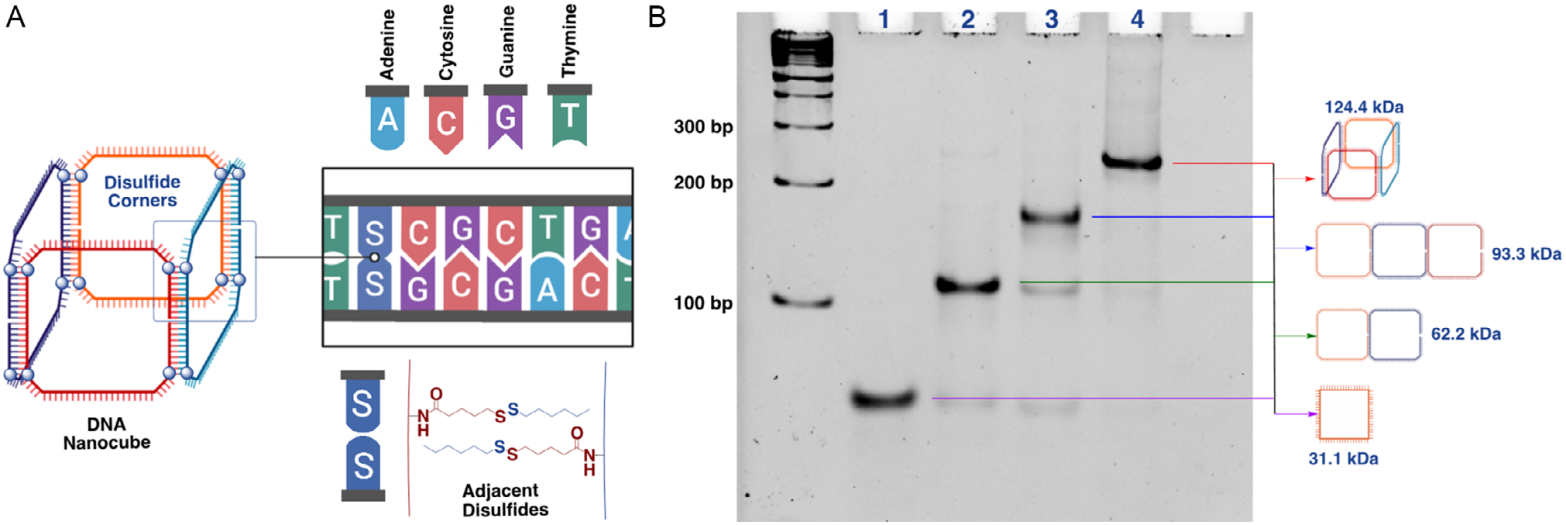
A) Schematic representation of disulfide corners on DNA nanocube. B) 6% native PAGE in 1x TAMg (Tris‐Acetate‐Magnesium) buffer displaying stepwise assembly of DNA nanocube. Lane (1): Clip 1; Lane (2): Clip 1 + Clip 2; Lane (3): Clip 1 + Clip 2 + Clip 3; Lane (4): Clip 1 + Clip 2 + Clip 3 + Clip 4. Migration of DNA occurs from cathode (−) at the top of the gel toward the anode (+) at the bottom of the gel.

Our group previously employed UV light irradiation to covalently crosslink DNA nanocubes.^[^ ^26^ ^]^ This involved strategically placing tetrathymidine repeats at the cube's corners, facilitating the formation of cyclopyrimidine dimers between proximal thymines and covalently linking the cube components together. While effective, the use of UV‐B light resulted in off‐target DNA damage or insufficient crosslinking yields when gentler irradiation methods were attempted. Another crosslinking strategy explored in our lab involves incorporating reducible disulfides into the hydrophobic core of SNAs and allowing interstrand crosslinks to form via dynamic covalent chemistry. However, because the disulfides aggregated primarily through the hydrophobic effect, achieving precise control over crosslinking was challenging, resulting in variability in crosslinking yields.^[^ ^39^ ^]^ In this work, we adapted elements of both strategies to achieve optimal crosslinking. Rather than relying on UV light to create covalent bonds between adjacent thymines, we designed a nanocube, having ≈7 nm sides, with single disulfide units precisely positioned at each of its corners. This ensures spatial proximity of disulfides (**Figure** **1**A) to template the efficient formation of interstrand crosslinks.

After designing and synthesizing the four component strands (“clips”) of the nanocube, we confirmed that the inclusion of disulfides did not interfere with the nanostructure's assembly. We characterized the stepwise assembly of the cube, one clip at a time, using native polyacrylamide gel electrophoresis (PAGE) in a buffer containing Mg^2+^ cations (Figure 1B). We observed the clean assembly of the four‐clip cube final product with near quantitative yields, indicating that the positioning of disulfide monomers at the corners did not disrupt the structure's assembly.

**Figure 2:**
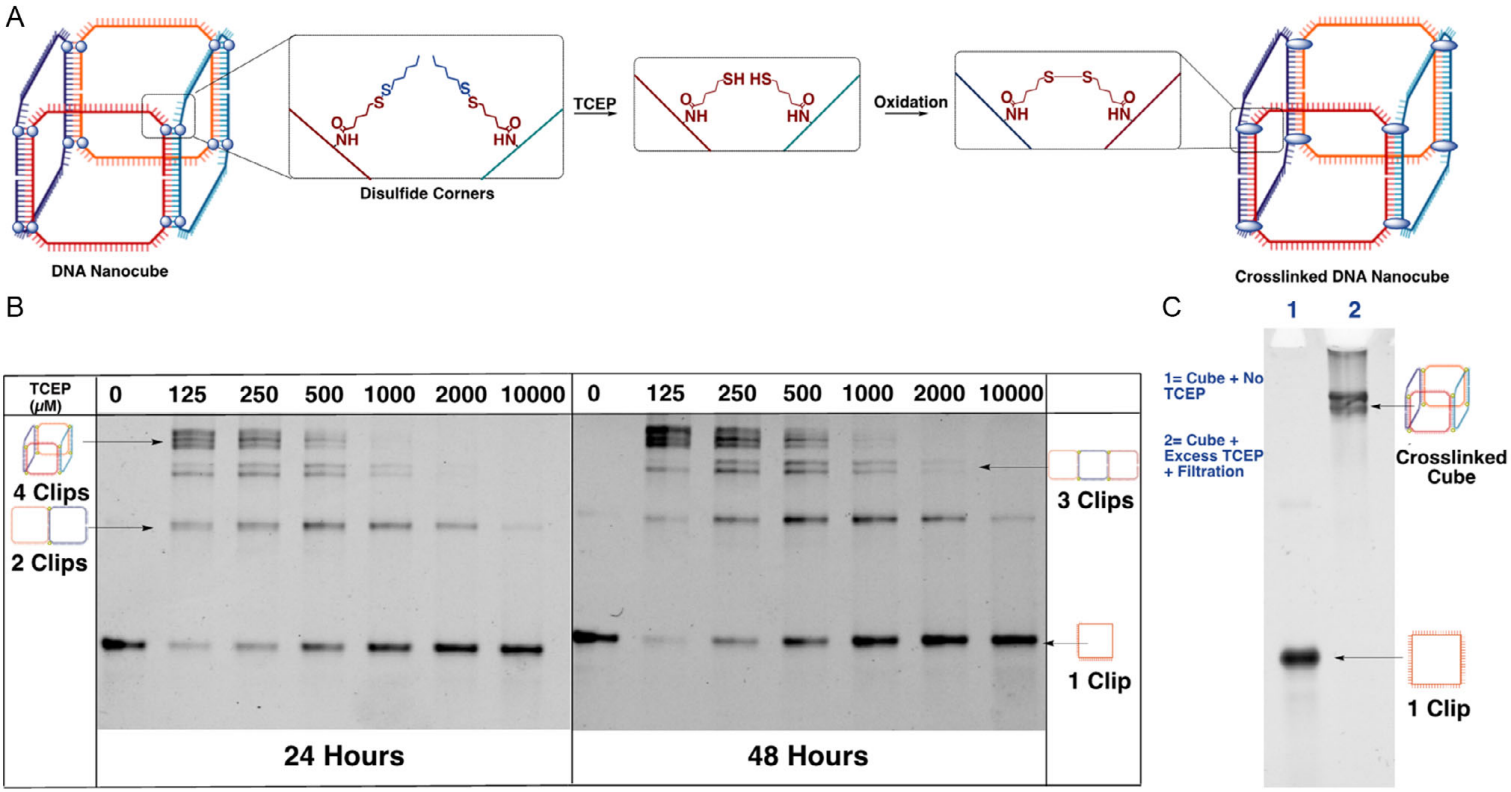
A) Schematic representation of crosslinking mechanism of DNA nanocube with TCEP as a reducing agent. B) 6% denaturing PAGE in 1x TBE (Tris‐Borate‐EDTA) buffer in 8 M urea upon incubation of 1 μM of DNA nanocube with varying concentrations of TCEP (0, 125, 250, 500, 1000, 2000 and 10 000 μM) for either 24 or 48 h. C) 6% denaturing PAGE in 1x TBE buffer in 8 M urea upon incubation of DNA nanocube with no TCEP (lane 1), or an excess of TCEP proceeded by amicon filtration (lane 2).

Next, we determined the necessary conditions for crosslinking of the disulfide units. We hypothesized that by reducing disulfide monomers into reactive thiols, their spatial proximity at the corners of adjacent clip strands would lead to the formation of interstrand disulfide crosslinks upon reoxidation (**Figure** **2**A). To test this hypothesis, we treated the assembled cube with different concentrations of TCEP (tris(2‐carboxyethyl)phosphine), a reducing agent. We incubated the samples for 24 and 48 h, and characterized the degree of crosslinking using denaturing PAGE, under nonreducing conditions (Figure 2B). The best yield of the four‐clip crosslinked cube (top band in the gel) was obtained upon incubation with 125 μM TCEP for 48 h (70%, Figure S2, Supporting Information). We also notice that the three‐“clip” crosslinked trimer and the fully crosslinked cube appear as several bands on the denaturing gel rather than one discrete band. This is due to cases of incomplete crosslinking that occur at the interface between every two complementary sides of the cube. This could allow for the formation of slightly different conformations of crosslinked product that have minorly shifted mobilities under denaturing conditions. While a structure may have one or more corners with incomplete crosslinking, it is still expected to show stability to denaturation and to nucleases. Incubation with TCEP at higher concentrations of nanocube can result in the formation of some mis‐formed and concatenated structures. We observe that this can be minimized by avoiding high concentrations of nanocube while crosslinking.

Interestingly, as the concentration of TCEP increased, we observed a decrease in the fully crosslinked four‐clip product, accompanied by an increase in the intermediate dimer and trimer crosslinks. In reducing conditions, the disulfides are converted to thiols and remain in this reduced state in the presence of an excess of TCEP, preventing crosslinking. In contrast, with less TCEP, the disulfides are reduced into thiols, but gradual aerobic oxidation with time ensures that the thiols reoxidize and crosslink the cube. At 48 h, we observed more extensive crosslinking than at 24 h for the same TCEP concentration, likely due to the oxidation of TCEP and the loss of reducing conditions, allowing for the formation of interstrand crosslinks.

To confirm this mechanism, we incubated our assembled nanocube with an excess of TCEP for 1 h to ensure complete reduction of disulfide monomers into thiols. We then filtered out the excess reducing agent to promote the formation of the final disulfide product by aerobic oxidation over 1 h. When this sample was characterized on a denaturing PAGE gel, we observed conversion into the fully‐crosslinked final product (95% yield, Figure S2, Supporting Information) without any intermediate dimers and trimers (Figure 2C).

### 2.2 Retaining Structural Integrity

After confirming successful nanocube crosslinking, the next step was to assess its structural integrity compared to the non‐crosslinked variant. A major limitation of DNA nanostructures in physiological applications is their reliance on high salt concentrations^[^ ^19^ ^]^ to counteract electrostatic repulsion and maintain assembly. Without sufficient salt concentration, specifically divalent cations, disassembly of the nanostructure occurs over time.^[^ ^40^ ^]^ By introducing disulfide crosslinks between adjacent clip sequences, our structure is stabilized by covalent bonds rather than just intermolecular DNA base pairing. This ensures structural integrity even in environments with accumulating negative charges and low concentrations of divalent cations.

**Figure 3:**
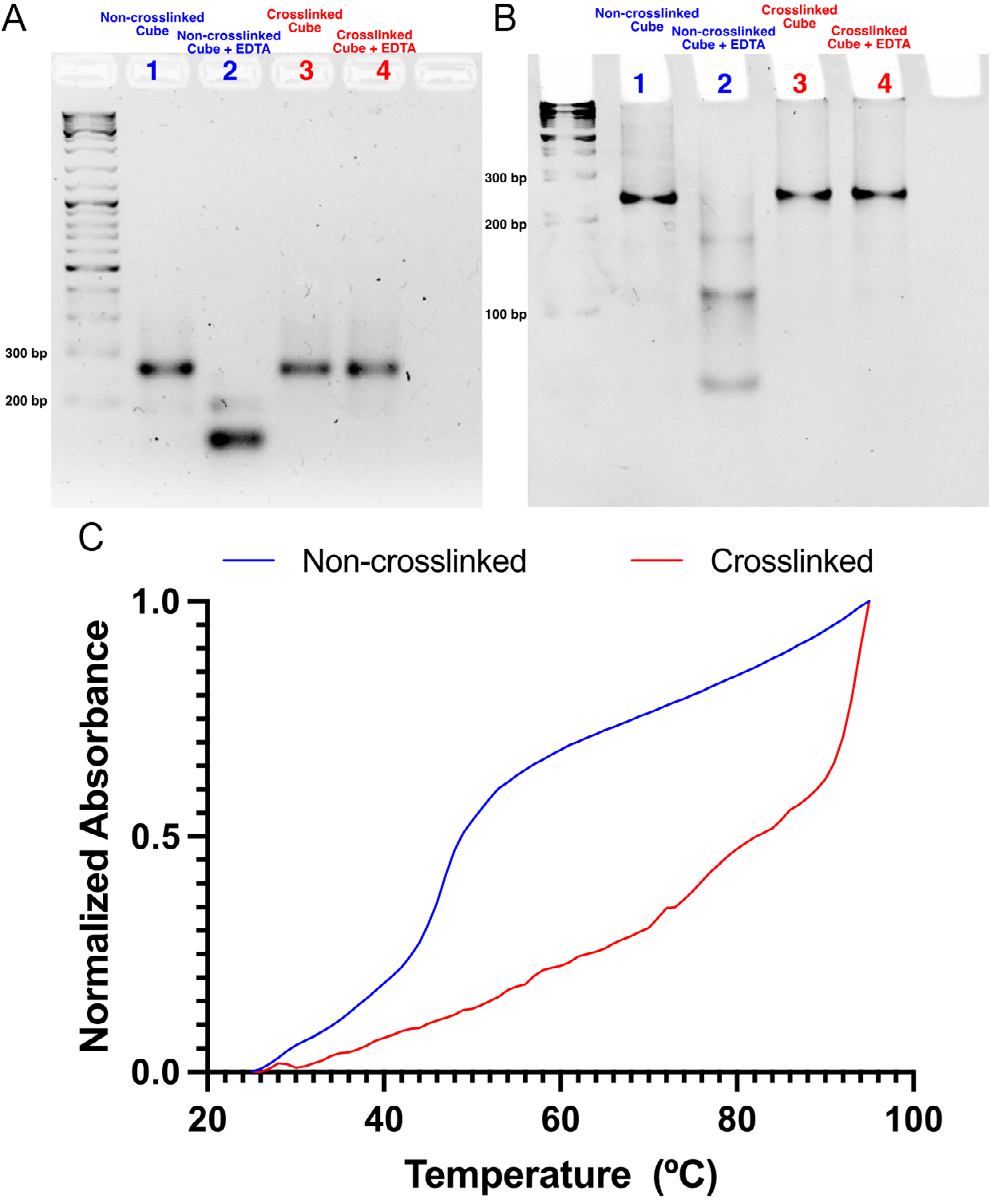
A) 2% AGE in 1x TAE (Tris‐Acetate‐EDTA) buffer or B) 6% native PAGE in 1x TAMg buffer containing non‐crosslinked cube and crosslinked cube (lanes 1 and 3, respectively) followed by incubation with an excess of EDTA (lanes 2 and 4, respectively). C) Thermal denaturation of non‐crosslinked (blue) and crosslinked (red) nanocubes by measuring absorbance at 260 nm as temperature was increased from 25 °C to 95 °C.

To test for this improved stability, we assembled both non‐crosslinked and crosslinked nanocubes under the same ionic conditions in a magnesium‐containing buffer, followed by the addition of excess ethylenediaminetetraacetic acid (EDTA), a chelating agent that sequesters divalent cationic species such as Mg^2+^ and Ca^2+^. We then characterized the structures with both agarose gel electrophoresis (AGE) in a magnesium‐free buffer (**Figure** **3**A) and PAGE in a buffer containing magnesium (Figure 3B), both before and after the addition of EDTA. We observed the maintenance of assembly for the crosslinked nanocube despite coincubation with EDTA, while the non‐crosslinked variant was disassembled into individual clip components. This validates that covalent crosslinking of the nanocube prevents structure disassembly in low salt concentrations, suggesting that the structure will remain assembled in physiologically relevant conditions.

Another concern with DNA nanostructures is the possibility of denaturation or melting at physiological temperatures (37 °C). Therefore, identifying the melting temperatures of both crosslinked and non‐crosslinked nanocube variants is important. To do so, the absorbance of DNA nanocubes, that were both assembled in the same magnesium‐containing buffer, was monitored at 260 nm as the temperature was gradually increased from 25 °C to 95 °C (Figure 3C). For the non‐crosslinked nanocube, a sharp thermal denaturation transition at 45 °C was observed, while the crosslinked nanocube retained assembly at higher temperatures, reaching ≈75 °C before melting—a 30 °C increase in melting temperature. This result was further confirmed by analyzing the heated constructs using native PAGE, where we observed partial dissociation of the non‐crosslinked cube into its components, while the crosslinked cube maintained its structure (Figure S4, Supporting Information). This improved thermal stability of the crosslinked nanocube is due to the covalent linkages between DNA duplexes in the clip sequences. These linkages preorganize the strands to bind even if base pairing is disrupted at high temperatures,^[^ ^41^ ^]^ resulting in an enhanced melting temperature (*T*_m_). These experiments confirmed the much higher stability of the crosslinked nanocube in biologically relevant conditions.

### 2.3 Interaction with Serum Proteins

When evaluating DNA nanostructures for biological applications, it is important to consider their interactions with serum proteins. This typically results in a protein corona that significantly impacts structural assembly and in vivo functionality.^[^ ^42^, ^43^ ^]^ While preferentially binding a specific protein may be advantageous in some cases,^[^ ^44^, ^45^ ^]^ this should not lead to structural disassembly. Previous reports show that self‐assembled nanostructures, such as SNAs, can disassemble upon binding prevalent serum proteins such as albumin, leading to structural loss and reduced gene silencing ability.^[^ ^39^, ^46^ ^]^

**Figure 4:**
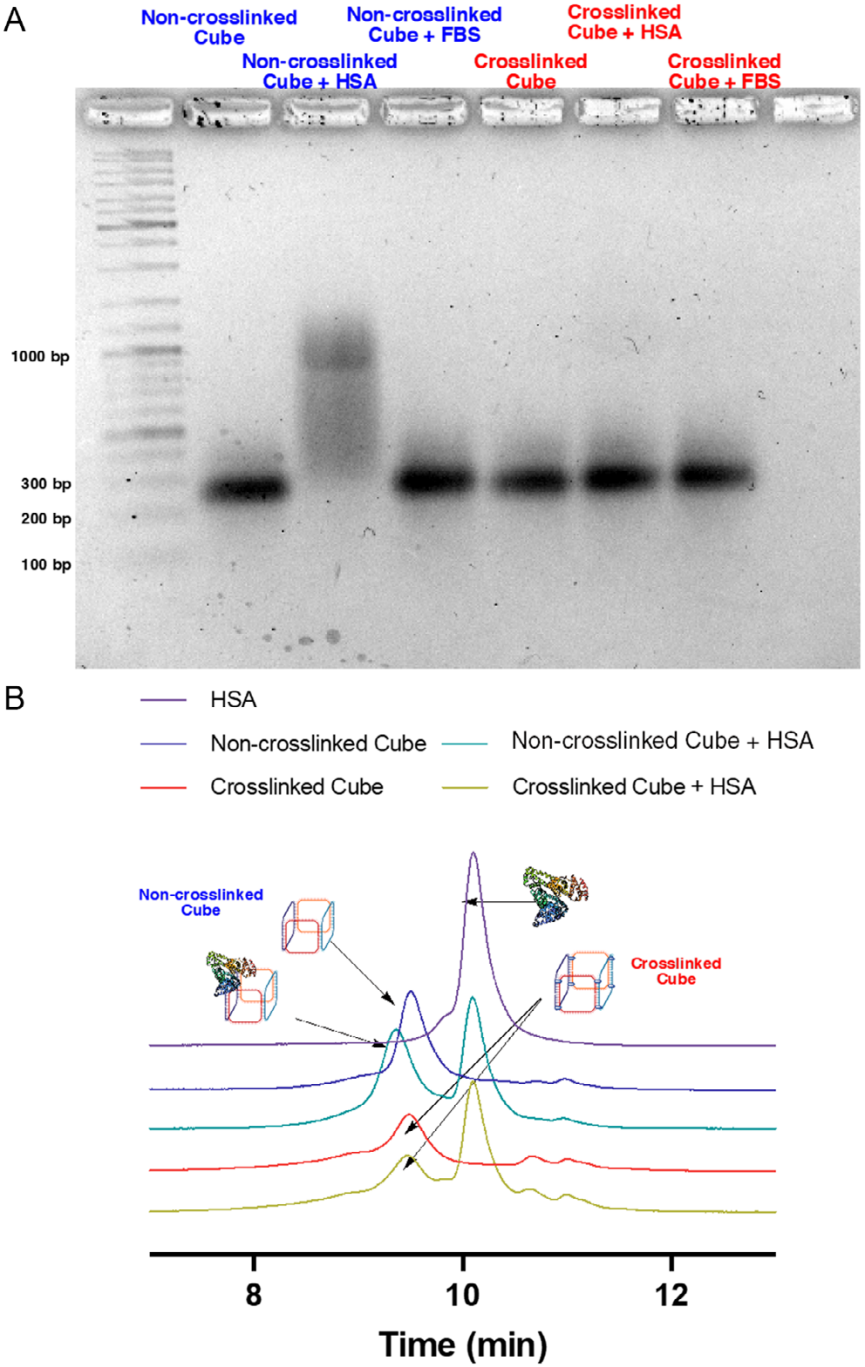
A) 2% AGE in 1x TAMg buffer of non‐crosslinked cube (blue) or crosslinked cube (red) after incubation with HSA or 10% FBS‐doped media. B) Size exclusion chromatogram in 1x TAMg displaying retention times of HSA and the non‐crosslinked and crosslinked cube, with and without preincubation in 75 μM of HSA.

To determine the interaction of our constructs with serum proteins, we performed an electrophoresis mobility shift assay (EMSA). In this experiment, our assembled nanostructures were incubated with human serum albumin (HSA) or 10% of fetal bovine serum (FBS) in cell culture medium (DMEM). Interestingly, we observed a shift to lower mobility when the non‐crosslinked nanocube is incubated with HSA, suggesting interaction with serum proteins (**Figure** **4**A). This can be explained by the presence of alkyl chains on the disulfide units in the non‐crosslinked cube (Figure 2A) and their interaction with albumin, a fatty acid‐binding protein. Upon reduction during disulfide crosslinking, these alkyl chains are cleaved off, significantly reducing the affinity of albumin to the cube. Importantly as well, neither structure displays a mobility shift after being incubated with FBS suggesting structure maintenance in physiologically relevant media.

To further examine the cube's interaction with serum proteins, we used size exclusion chromatography. Upon incubating our samples with HSA, the non‐crosslinked cube shifts to a lower retention time (Figure 4B), indicating the formation of a larger construct due to binding with albumin. In contrast, the crosslinked cube maintains the same retention time with and without HSA reflecting its low affinity to albumin. This agrees with the EMSA results which suggest that the non‐crosslinked structure does not interact with nor form a larger complex with HSA (Figure 4A). Both cube variants remain intact, but likely display different protein coronas due to different interactions with serum proteins.

### 2.4 Degradation by Nucleases

DNA is naturally degraded in the body by enzymes known as nucleases, which challenges the use of DNA nanostructures in biological applications.^[^ ^47^, ^48^ ^]^ Since our DNA nanocubes are made of regular unmodified nucleotides—therefore sensitive to nuclease degradation—determining the lifetime of our structures in nuclease‐containing environments is essential.

**Figure 5:**
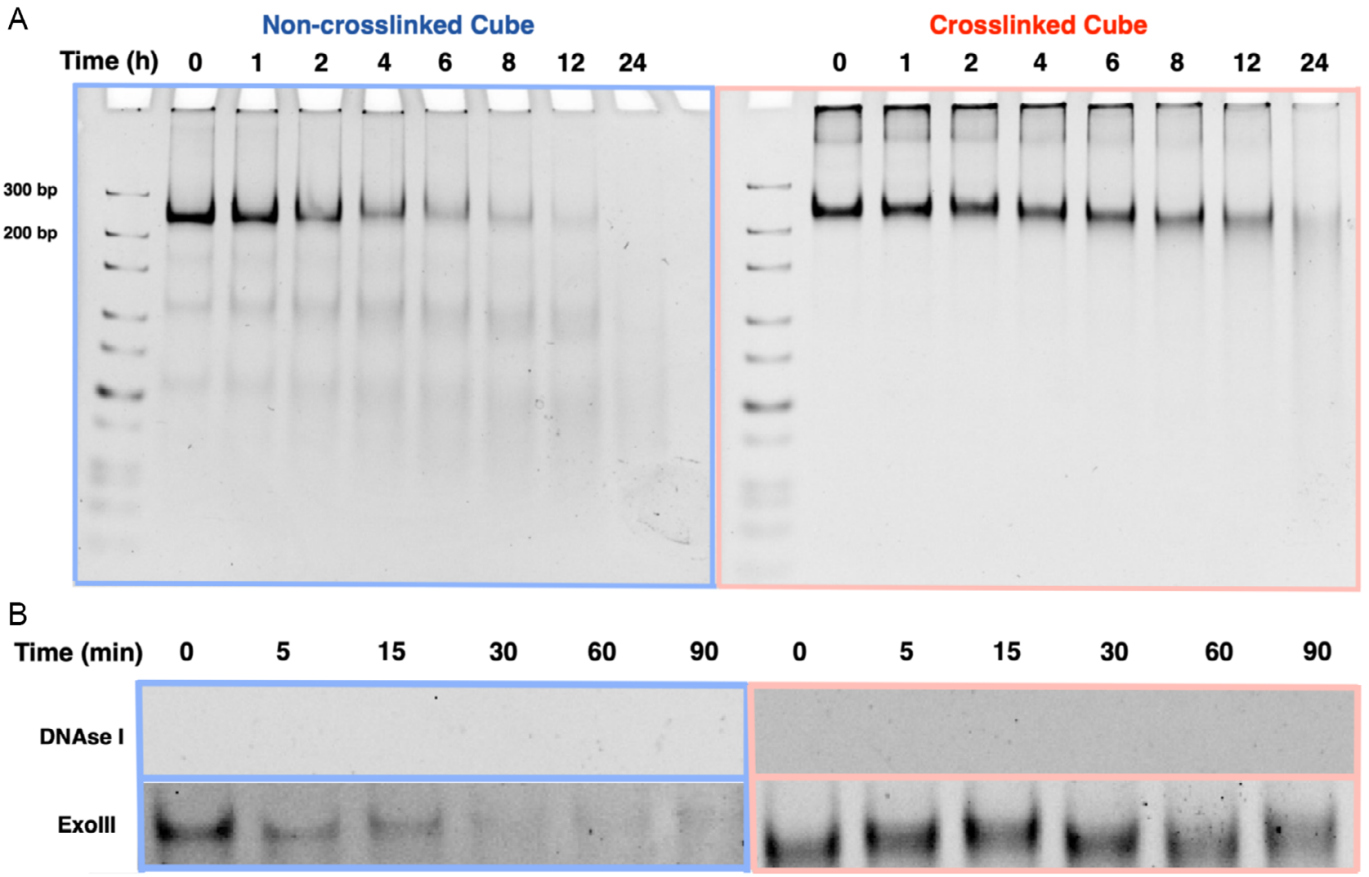
A) 6% native PAGE in 1x TAMg of non‐crosslinked cube (blue) and crosslinked cube (red) incubated in 10% FBS‐doped DMEM for 0, 1, 2, 4,6, 8, 12, and 24 h. B) 6% native PAGE of non‐crosslinked cube (blue) and crosslinked cube (red) incubated in DNAseI, ExoI, and ExoIII for 0, 5, 15, 30, 60, and 90 min.

To evaluate the stability of the constructs to nucleases, non‐crosslinked and crosslinked nanocubes, preassembled under the same ionic conditions, were incubated in DMEM + 10% FBS, and aliquots were taken at different times. Gel electrophoresis was used to quantify the rate of degradation. The non‐crosslinked cube immediately degraded into subproducts, while the crosslinked nanocube maintained its structure with minimal degradation for up to 6 h (**Figure** **5**A). After 24 h of incubation, both constructs completely degraded. Thus, covalent crosslinking provides significant, albeit partial protection from nuclease degradation. These observations can mainly be explained due to the presence of two types of nucleases present in serum: exonucleases which initiate DNA degradation from either the 3′ or 5′ ends of a sequence, or endonucleases which can internally degrade nucleic acid sequences. The improved stability of the crosslinked cube in serum is likely due to enhanced exonuclease resistance. However, we hypothesized that prolonged endonuclease activity is what is leading to the degradation of both structures with time.

To validate these hypotheses, we incubated the structures individually with specific nucleases. First, both structures were incubated with DNAseI, an endonuclease that nonspecifically cleaves both double‐ and single‐stranded DNA. The enzyme rapidly degraded both structures (Figure 5B). This outcome is expected since the nanocubes are composed of unmodified DNA which is easily recognized by the enzyme. Thus, the degradation of our structures upon long‐term serum incubation is likely the result of endonuclease activity.

With ExoI, an exonuclease that specifically cleaves single‐stranded DNA from the 3′ to 5′ position, we observed no degradation of either structure, consistent with the absence of single‐stranded terminal DNA regions (Figure S7, Supporting Information). Finally, we tested ExoIII, an exonuclease that degrades double‐stranded DNA from the 3′ to 5′ end. The non‐crosslinked structure rapidly degraded into subproducts, while the crosslinked cube remained intact (Figure 5B), even at higher enzyme concentrations (Figure S6, Supporting Information). This stability is likely due to the covalent linkages at the cube's corners, which block the enzyme's progress as it degrades the 3′ terminus of each clip sequence. In contrast, the non‐crosslinked structure lacks these linkages, allowing ExoIII to fully degrade the cube. Covalently crosslinking the corners of the cube therefore results in a prolonged serum stability due to increased exonuclease resistance.

### 2.5 Cellular Uptake and Inhibition

After demonstrating that the crosslinked nanocube displays greater serum stability than the non‐crosslinked construct, our next goal was to determine whether this improvement leads to enhanced cellular uptake. We focused on the first 6 h, where the most significant difference in serum stability was observed. To track uptake, we synthesized a DNA sequence complementary to a single‐stranded face of the cube, labeled with a non‐cell permeable fluorescent sulfo‐Cyanine 3 dye (Table S1, Supporting Information). This sequence was fully modified with 2′‐fluoro and 2′‐methoxy nucleotides to prevent its degradation and detachment from the nanocube, which could lead to a false positive signal.^[^ ^49^ ^]^

**Figure 6:**
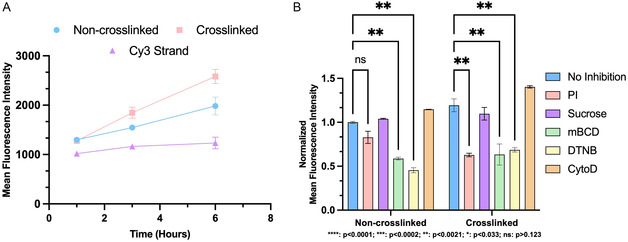
A) Measured fluorescence intensity in HeLa cells after incubation with 200 nM of suflo‐Cy3‐doped non‐crosslinked and crosslinked nanocubes, as well as individual Cy3 strand for 1, 3, and 6 h to demonstrate cellular uptake of different constructs with time. B) Measured fluorescence intensity in HeLa cells after incubation with 200 nM of suflo‐Cy3‐doped non‐crosslinked and crosslinked nanocube after coincubation with polyinosinic acid (PI) (500 μg mL^−1^), sucrose (15 mM), methyl‐β‐cyclodextrin (mBCD) (3 mM), 5,5′‐dithiobis‐(2‐nitrobenzoic acid) (DTNB) (1.2 mM), or cytochalasin D (CytoD) (10 μM). Error bars represent SD of duplicate experiments for each sample. ****p < 0.0001; ***p < 0.0002; **p < 0.0021; *p < 0.033; ns: *p* > 0.123.

First, we wanted to evaluate the cytotoxicity of our constructs, so we preassembled our non‐crosslinked and crosslinked nanocubes, then incubated them in HeLa cells for 24 h with and without the use of transfection agents. Upon doing so, no cytotoxicity was observed for either construct (Figure S12, Supporting Information). Next, HeLa cells were incubated with the dye‐labeled constructs, and separately, with the single‐stranded DNA probe, for 1, 3, and 6 h at 37 °C. Cellular uptake was measured by flow cytometry (**Figure** **6**A and Figure S10, Supporting Information) after washing the cells to remove extracellular and membrane‐bound DNA. Across all time points, both nanocube variations exhibit significantly higher cellular uptake than the single‐stranded sequence. This is likely due to the ability of these larger, densely negatively charged, DNA nanostructures to activate additional endocytosis pathways.^[^ ^13^, ^50^, ^51^, ^52^ ^]^

Initially, after 1 h of incubation, both non‐crosslinked and crosslinked nanocubes display similar degrees of cell penetration. However, at 3 and 6 h, the crosslinked cube displays a 20% and 30% increase in cellular uptake respectively, likely due to its structural integrity in serum. This difference becoming more pronounced over time, likely reflects the crosslinked cube's sustained stability.

To verify the mechanisms of cellular uptake of these compounds, an inhibition study was performed at the 3 h time point, where differences in uptake between the constructs start to become more noticeable. Before adding the DNA structures, the cells were preincubated for 30 min with inhibitors targeting specific uptake pathways: polyinosinic acid^[^ ^53^ ^]^ (PI, scavenger receptor inhibitor), sucrose^[^ ^54^ ^]^ (clathrin‐mediated endocytosis inhibitor), methyl‐β‐cyclodextrin^[^ ^55^, ^56^ ^]^ (mBCD‐, caveolin‐, and clathrin‐mediated endocytosis inhibitor), 5,5′‐dithiobis‐(2‐nitrobenzoic acid)^[^ ^57^ ^]^ (DTNB, thiol‐mediated uptake inhibitor), and cytochalasin D^[^ ^58^ ^]^ (CytoD, macropinocytosis and phagocytosis inhibitor).

For both non‐crosslinked and crosslinked cubes, no major decrease in uptake was observed for inhibition with CytoD, suggesting neither macropinocytosis nor phagocytosis is involved in uptake. However, upon incubation with polyinosinic acid, a partial decrease in the non‐crosslinked cube uptake was observed, and a significantly larger decrease (18% vs 48%) in crosslinked cube uptake is observed (Figure 6B and Figure S11, Supporting Information). This demonstrates that the enhanced uptake of the crosslinked cube is primarily due to increased scavenger receptor‐mediated endocytosis. The crosslinked cube's ability to maintain its structure likely contributes to this uptake mechanism; in contrast, the non‐crosslinked cube degrades into subproducts over time that may not trigger the same uptake pathway.

Moreover, both non‐crosslinked and crosslinked constructs are inhibited by mBCD, but not by sucrose. Since neither construct is inhibited by sucrose, responsible for clathrin uptake inhibition, this suggests that caveolin‐mediated endocytosis is likely involved in their mechanism of uptake. Inhibition by DTNB is more pronounced for the non‐crosslinked cube than the crosslinked one, which indicates a higher involvment of thiol‐mediated uptake pathways for the former.^[^ ^59^ ^]^ This is expected as the disulfide moieties in the non‐crosslinked cube are more accessible to react with thiols at the cell surface, the alkyl chain potentially interacting with the plasma membrane. In both cases, the interaction of the cube with scavenger receptors at the cell surface could also help involving thiol‐mediated pathways, as shown for disulfide‐containing DNA origami constructs^[^ ^60^ ^]^ and demonstrated here by DTNB inhibition.

Overall, these experiments showed that the increased stability of the DNA nanocube upon disulfide crosslinking translated to an improved ability to penetrate cells by better engaging scavenger receptors. This results in caveolin‐mediated endocytosis, with participation of thiol‐mediated uptake pathways, showing the potential of this method to greatly overcome a major bottleneck in the biological applications of DNA wireframe nanostructures.

## 3 Conclusion

In summary, we developed a simple method to crosslink DNA nanocubes using strategically positioned disulfide monomers and dynamic covalent chemistry, resulting in nearly quantitative crosslinking yields. This approach significantly enhanced structural stability and cellular uptake, addressing key challenges for physiological applications.

We systematically assessed stability parameters relevant to biological use. Crosslinked structures maintained their structural integrity in environments deprived of divalent cations, unlike non‐crosslinked nanocubes which dissociated. The crosslinked structures also exhibited a 30 °C increase in melting temperature, indicating significantly enhanced thermal stability. Both DNA nanocubes did not dissociate upon interacting with serum proteins such as albumin. Notably, the crosslinked structure demonstrates increased resistance to nuclease degradation in serum, particularly due to its enhanced stability. This increased serum stability correlated with greater cellular penetration for the crosslinked cube. Inhibition studies identified that uptake occurs via interaction with scavenger receptor‐mediated endocytosis as a key contributor to cellular uptake.

We anticipate that this site‐specific crosslinking approach can be adapted to other DNA nanostructures, enabling high‐yield, selective covalent linking of DNA‐based materials. Further work will investigate enhancing the resistance of these structures against endonucleases. Once optimized, these structures could achieve long‐term stability, enabling therapeutic applications such as gene silencing in both in vitro and in vivo settings.

## Conflict of Interest

The authors declare no conflict of interest.

## Author Contributions

**Sinan Faiad**: conceptualization (supporting); formal analysis (lead); investigation (lead); methodology (equal); visualization (lead); writing—original draft (equal); writing—review and editing (equal). **Quentin Laurent**: conceptualization (supporting); formal analysis (supporting); investigation (supporting); methodology (equal); supervision (equal); writing—original draft (equal); writing—review and editing (lead). **Jathavan Asohan**: investigation (supporting); methodology (supporting); writing—review and editing (supporting). **Tyler Brown**: conceptualization (supporting); investigation (supporting); methodology (supporting); writing—review and editing (supporting). **Alexander Prinzen**: conceptualization (supporting); investigation (supporting); methodology (supporting). **Hanadi Farouk Sleiman**: conceptualization (supporting); funding acquisition (lead); project administration (lead); resources (lead); supervision (lead); writing—review and editing (equal).

## Supporting information

Supplementary Material

## Data Availability

The data that support the findings of this study are available from the corresponding author upon reasonable request.
