# Supplementary material for: Site‐Specific Disulfide‐Mediated Crosslinking of DNA Nanocubes for Enhanced Biological Applications
Source: Small Sci. 2024 Dec 12;5(4):2400471. doi: 10.1002/smsc.202400471 (PMC12244501; doi:10.1002/smsc.202400471)
Supplement: Supplementary file 1 — Supplementary Material [file SMSC-5-2400471-s001.pdf]

Supporting Information

Site-Specific Disulfide-Mediated Crosslinking of DNA Nanocubes for Enhanced Biological Applications

Sinan Faiad, Quentin Laurent, Jathavan Asohan, Tyler Brown, Alexander Prinzen, and Hanadi F. Sleiman*

Department of Chemistry, McGill University

801, Sherbrooke St. West, Montreal, QC, Canada, H3A 0B8

*E-mail: hanadi.sleiman@mcgill.ca

Table of Contents

1. General 3
2. Instrumentation 4
3. Synthesis of Phosphoramidites 5
4. Solid-Phase Synthesis 5
5. Gel Purification 6
6. HPLC Purification 6
7. LC-ESI-MSI Characterization 7
8. Gel Electrophoresis Analysis 10
9. Cube Crosslinking Optimization 11
10. Dynamic Light Scattering 12
11. Thermal Denaturation 13
12. Electrophoretic Mobility Shift Assay (EMSA) 14
13. Size Exclusion Chromatography (SEC) 15
14. Nuclease Cleavage Assays 15
15. Serum Stability 19
16. Flow Cytometry and Cell Inhibition 19
17. Cellular Cytotoxicity 22
18. Supporting References 23

1. General

All starting materials were obtained from commercial suppliers and used without further purification unless otherwise noted. Tris(hydroxymethyl)aminomethane (Tris), ethylenediaminetetraacetate (EDTA), urea, 40% acrylamide/bis-acrylamide (19:1), ammonium persulfate (APS), N,N,N',N'- tetramethylethane-1,2 diamine (TEMED) and agarose were purchased from BioShop Canada Inc and used without further purification. Magnesium chloride hexahydrate was purchased from Sigma-Aldrich. Acetic acid, boric acid, ammonium hydroxide and 10x PBS (with magnesium, calcium) were purchased from Fisher Scientific and used without further purification. Red Nucleic Acid Gel Stain was purchased from MedChemExpress. GeneRuler DNA Ladder Mix and DNA Gel Loading Dye (6X) were obtained from Thermo Scientific. 1 µmol 1000 Å universal synthesis CPG column, standard reagents used for automated DNA synthesis, N,N-diisopropylamino Cyanoethyl phosphonamidic-chloride (CEP-Cl), 2’-fluoro 2’-deoxy CED phosphoramidites (cat.# ANP-9151, ANP-9152, ANP-9154, and ANP-9159), and 2’-O-Methyl CED phosphoramidites (cat.# ANP-5751, ANP-5752, ANP-5754, ANP-6759) were purchased from Chemgenes. Sulfo-Cyanine 3 azide was purchased from Lumiprobe (cat.# A1330). Sulfurizing Reagent II (cat.# 40-4037) and 5’ DBCO-TEG Phosphoramidite (cat.# 10-1941-90) were purchased from Glen Research. DMT-Cl was purchased from AK Scientific. Starting materials for phosphoramidite synthesis were purchased from Oakwood Chemicals. Dulbecco’s Modified Eagle Medium (DMEM) and phosphate-buffered saline (PBS) were purchased from Life Technologies. Lipofectamine reagent was purchased from Invitrogen. Fetal bovine serum (FBS), 0.05% Trypsin-EDTA and sodium pyruvate were obtained from Wisent Bioproducts. The Bright-Glo Luciferase Assay system was purchased from Promega. Cell-Titer Blue assay was also purchased from Promega. The HeLa X1/5 cells stably expressing luciferase were purchased from Millipore Sigma (95051229-1VL).

All other reagents were obtained from Sigma-Aldrich. TEAA buffer is composed of 50mM TEA with pH adjusted to 8.0 using glacial acetic acid. TBE buffer is 90 mM Tris, 90 mM boric acid and 1.1 mM EDTA with a pH of 8.0. TAMg buffer is 40 mM Tris, 7.6 mM magnesium chloride and 1.4 mM acetic acid. TAE buffer is 40 mM Tris, 20 mM acetic acid, and 1 mM EDTA.

Abbreviations. ACN: Acetonitrile; AGE: Agarose gel electrophoresis; APS: Ammonium persulfate; CPG: Controlled pore glass; Cy3: Cyanine 3 Dye; DIPEA: Diisopropylethylamine; DMEM: Dulbecco's Modified Eagle Medium; DMT: Dimethoxytrityl; EDTA: Ethylenediaminetetraacetate; ESI-MS: Electrospray ionization mass spectrometry; ETT: 5-(ethylthio)-1H-tetrazole; FACS: Fluorescence-activated cell sorting; FBS: Fetal bovine serum; HATU: Hexafluorophosphate azabenzotriazole tetramethyl uronium; HSA: Human serum albumin; HPLC: High-pressure liquid chromatography; LC: Liquid chromatography; MS: Mass spectrometry; PAGE: Polyacrylamide gel electrophoresis; PBS: Phosphate-buffered saline; PDI: Polydispersity index; PK: Proteinase K; QTOF: Quadrupole time-of-flight; RP: Reverse phase; SEC: Size exclusion chromatography; TAE: Tris acetic acid EDTA; TAMg: Tris acetic acid magnesium chloride; TBE: Tris boric acid EDTA; TCEP: tris(2-carboxyethyl)phosphine; TEA: Triethylamine; TEAA: Triethylammonium acetate; Tetrathiol **1**: pentaerythritol tetra(3-mercaptopropionate) TEMED: Tetramethylethylenediamine; TLC: Thin layer chromatography; Tris: Trisaminomethane.

2. Instrumentation

Standard oligonucleotide synthesis was performed on solid supports using a Mermade MM6 synthesizer from Bioautomation. UV absorbance DNA quantification measurements were performed with a NanoDrop One-C spectrophotometer from Thermo Scientific. For structure assembly, Eppendorf Mastercycler 96-well thermocycler and Bio-Rad T100TM thermal cycler were used to anneal all DNA nanoparticles. Polyacrylamide gel electrophoresis (PAGE) was performed using 20x20 cm vertical Hoefer 600 electrophoresis units, or a Bio-Rad Mini-PROTEAN® Tetra Vertical electrophoresis units. Agarose gel electrophoresis (AGE) was performed on Owl Mini and Owl EasyCast horizontal gel systems. Gels were imaged by BioRad ChemiDoc MP or XR+ systems. Liquid Chromatography Electrospray Ionization Mass Spectrometry (LCESI-MS) was carried out using Dionex Ultimate 3000 coupled to a Bruker MaXis Impact™ QTOF. Size exclusion chromatography was performed on an Agilent 1260 Infinity II HPLC equipped with an Agilent AdvancedBio SEC column (2.7 µm, 300 mm, 300 Å, flow rate 0.3 mL/min of 1x TAMg, 12 min). Column chromatography to purify organic

compounds was performed on a CombiFlash® Rf + system with RediSep® Silica columns (230-400 mesh). ^{1}H NMR, ^{13}C and ^{31}P were recorded on 500 MHz AV500 equipped with a 60 position SampleXpress sample changer (Bruker) and 300 MHz Varian Mercury equipped with an SMS-100 sample changer (Agilent). Visualization of TLC was achieved by UV light (254 nm). Chemical shifts were quoted in parts per million (ppm) referenced to the appropriate residual solvent peak or 0.0 ppm for tetramethylsilane. Abbreviations for ^{1}H NMR: s = singlet, d = doublet, t = triplet, q = quartet, quint = quintet, m = multiplet. High-resolution mass spectra were obtained from Exactive Plus Orbitrap Mass Spectrometer (Thermo Scientific).

3. Synthesis of Phosphoramidites

Disulfide phosphoramidite was synthesized according to a reported procedure[1].

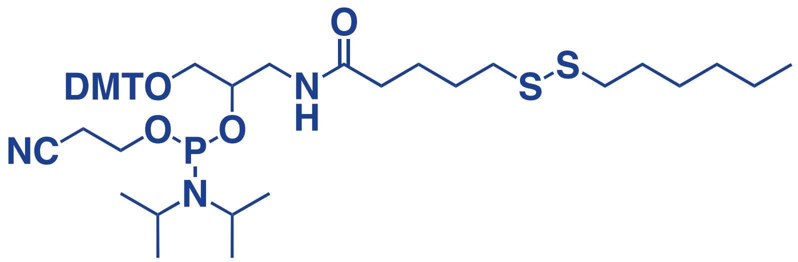

Scheme S1. Structure of chemically synthesized disulfide phosphoramidite.

4. Solid-Phase Synthesis

Standard oligonucleotide synthesis was performed on a 1 µmol scale, starting from the required 1000 Å Universal UnyLinker Solid Support purchased from Chemgenes. Coupling efficiencies were monitored by the removal of the DMT (dimethoxytrityl) on 5'-OH groups. In a glove box under an inert nitrogen atmosphere (<0.04 ppm for oxygen, and <0.5 ppm trace moisture), the disulfide phosphoramidite was dissolved in anhydrous acetonitrile to achieve a final concentration of 0.1 M. 2'-fluoro 2'-deoxy CED phosphoramidites (cat.# ANP-9151, ANP-9152, ANP-9154, and ANP-9159), and 2'-O-Methyl CED phosphoramidites (cat.# ANP-5751, ANP-5752, ANP-5754, ANP-6759) were purchased from Chemgenes and were also dissolved in anhydrous acetonitrile to achieve final concentrations of 0.1 M.

These compounds were then activated using 0.25 M 5-(ethylthio)tetrazole in anhydrous acetonitrile and an extended coupling time of 5 min was employed. 3% dichloroacetic acid in dichloromethane was used to remove the DMT protecting group on the DNA synthesizer. Oxidation was performed prior to capping on the synthesizer using 0.02M Iodine in Tetrahydrofuran/Water/Pyridine (70:20:10) (cat.# 40-4330, Glen Research). Alternatively, Sulfurization was executed prior to capping on the DNA synthesizer using Sulfurizing Reagent II (cat.# 40-4037, Glen Research). Following synthesis, oligonucleotide strands were cleaved from the solid support using 28% aqueous ammonium hydroxide solution for 36 h at rt. Strands were dried under vacuum at 40 ºC, resuspended in Millipore H_2O.

5. Gel Purification

Disulfide clip sequences were purified by PAGE after DNA synthesis. Aqueous solution of oligomer was mixed with an equal volume of Urea prior to loading on gel in order to assist in denaturation. oligomers were run on 12% polyacrylamide/8M urea gels in 1xTBE for 30 minutes at 250V, followed by 60 minutes at 500V. Following gel electrophoresis, bands were imaged using a handheld UV illuminator (254nm) and excised, crushed, and suspended in ~5-10 mL H_2O. This suspension was frozen by brief submersion in liquid nitrogen, before being incubated at 60°C for 16 hours. The supernatant was concentrated by evaporation, desalted using size exclusion chromatography (Sephadex G-25), and quantified (OD260) using a NanoDrop One-C spectrophotometer from Thermo Scientific.

6. HPLC Purification

Crude samples in 20 – 100 µL of Millipore water were injected into a Hamilton PRP-1 5 µm 2.1x150 mm column at 60 °C. The mobile phases were TEAA buffer at pH 8.0 and HPLC grade acetonitrile, with an elution gradient from 3 to 50% acetonitrile over 35 min. Strands were detected using a diode array detector monitoring absorbance at 260 nm as well as absorbance at 550nm for Cy3-labeled sequences.

7. LC-ESI-MS Characterization

The oligomers were analyzed by LC-ESI-MS in negative ionization mode using on a Dionex Ultimate 3000 UHPLC coupled to a Bruker Maxis Impact Q-TOF mass spectrometer.

Method 1: Samples (10 to 25 µM, 12 µL) were run through an Agilent AdvancedBio C_{18} column (2.1 x 50 mm, 120 Å, 2.7 µm) at a flow rate of 0.2 mL/min at 40 °C using a gradient of mobile phase A (100 mM 1,1,1,3,3,3-hexafluoro-2-propanol and 5 mM TEA in water) and mobile phase B (Methanol) in 8 min (2% to 100% B). Data was processed using the Bruker Data Analysis software version 4.2. *Method 2:* Samples (10 to 25 µM, 12 µL) were run through an Agilent Poroshell HPH-C_8 column (3.0 x 100 mm, 100 Å, 2.7 µm) at a flow rate of 0.3 mL/min at 60 °C using a gradient of mobile phase A (100 mM 1,1,1,3,3,3-hexafluoro-2-propanol and 5 mM TEA in water) and mobile phase B (1:1 methanol/acetonitrile) in 15 min (50% to 100% B). Data was processed using the Bruker Data Analysis software version 4.2.

Table S1. ESI-MS characterization of the oligomers.

Oligomer	Sequence[a]	Calculated MW [g mol^{-1}]	Found MW [g mol^{-1}][b]
**C1-SS**	TAGCTGAGTAT**X**TTTTCCTATATGGTCAACTGCTCTTT**X**T GCAAGTGTTGGAACGCACACT**X**TTTGTAGTAATACCAGA TGGAGTTTT**X**TCACAAATCTG	31119.07	31118.13
**C2-SS**	CAATCGGTAGT**X**TTTTCCTATATGGTCAACTGCTCTTT**X**T TACTCAGCTACAGATTTGTGT**X**TTTGTAGTAATACCAGAT GGAGTTTT**X**TCAACTAGCTG	31075.02	31075.16
**C3-SS**	CACTGGTCAGT**X**TTTTCCTATATGGTCAACTGCTCTTTXT CTACCGATTGCAGCTAGTTGT**X**TTTGTAGTAATACCAGAT GGAGTTTT**X**TGGTTTGCTTA	31089.99	31088.90
**C4-SS**	CAACACTTGCT**X**TTTTCCTATATGGTCAACTGCTCTTT**X**TC TGACCAGTGTAAGCAAACCT**X**TTTGTAGTAATACCAGAT GGAGTTTT**X**TGTGTGCGTTC	31021.98	31021.19
**FM-Cy3**	sCy3-DBCOacUCCAUCUGGUAUUACUaC	7738.32	7738.16

a) Uppercase: phosphate linkage; lowercase: phosphorothioated linkage; Yellow Highlight: 2'Fluoro-2'-deoxy sugar; Green Highlight: 2'-O-Methyl sugar; **X**: disulfide modification; DBCO: 5'-DBCO-TEG modification; sCy3: sulfo-Cyanine 3 azide click modification. b) Mass was found using ESI-MS technique, detecting multiply charged species.

C1-SS

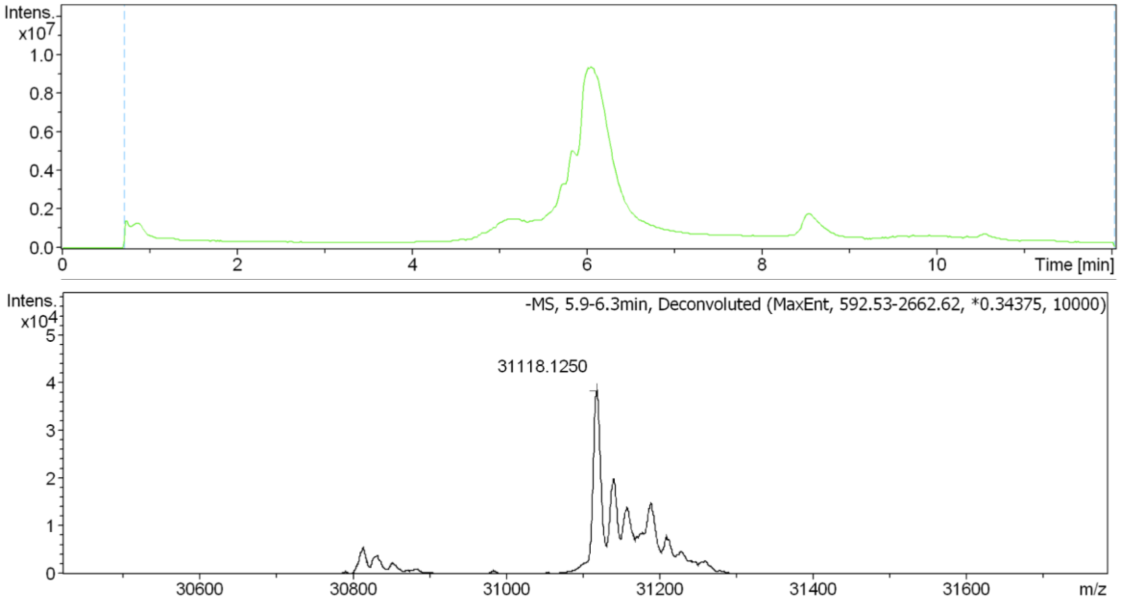

C2-SS

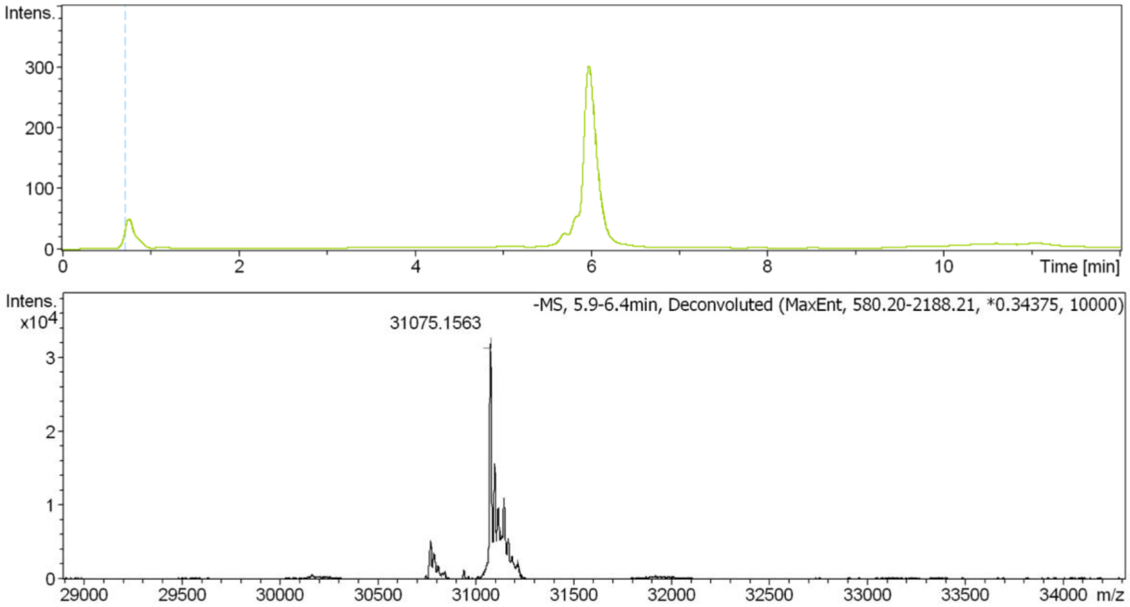

Figure S1. RP-HPLC traces of modified oligonucleotide strands (UV detection, 260 nm) and MS characterization.

C3-SS

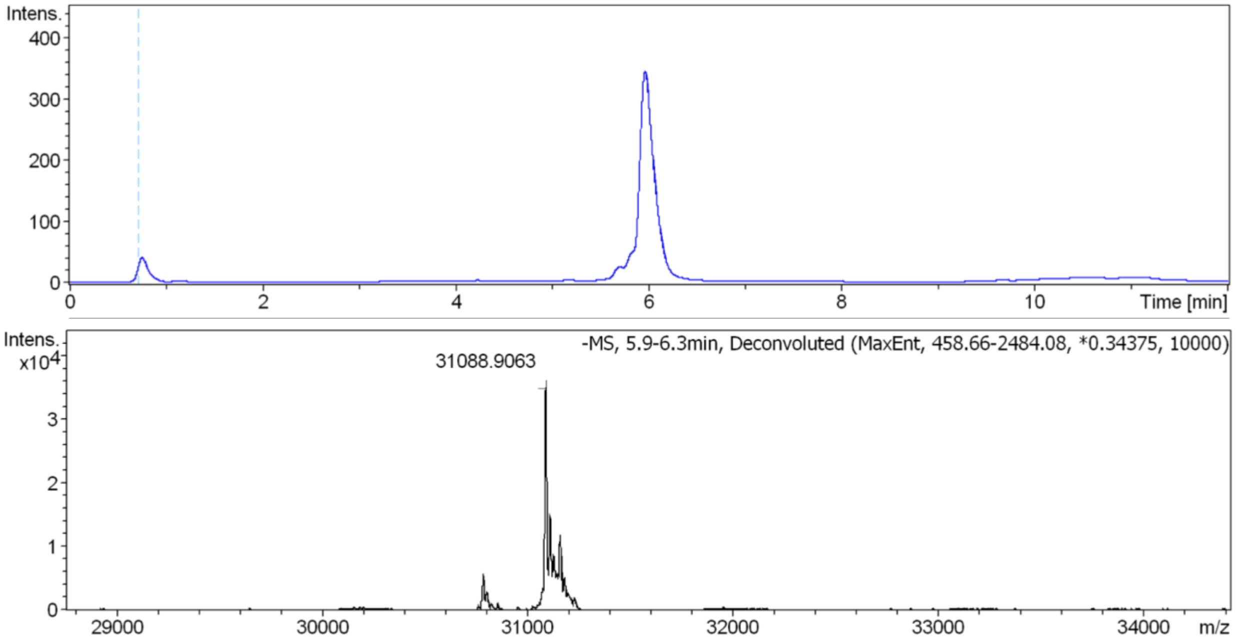

C4-SS

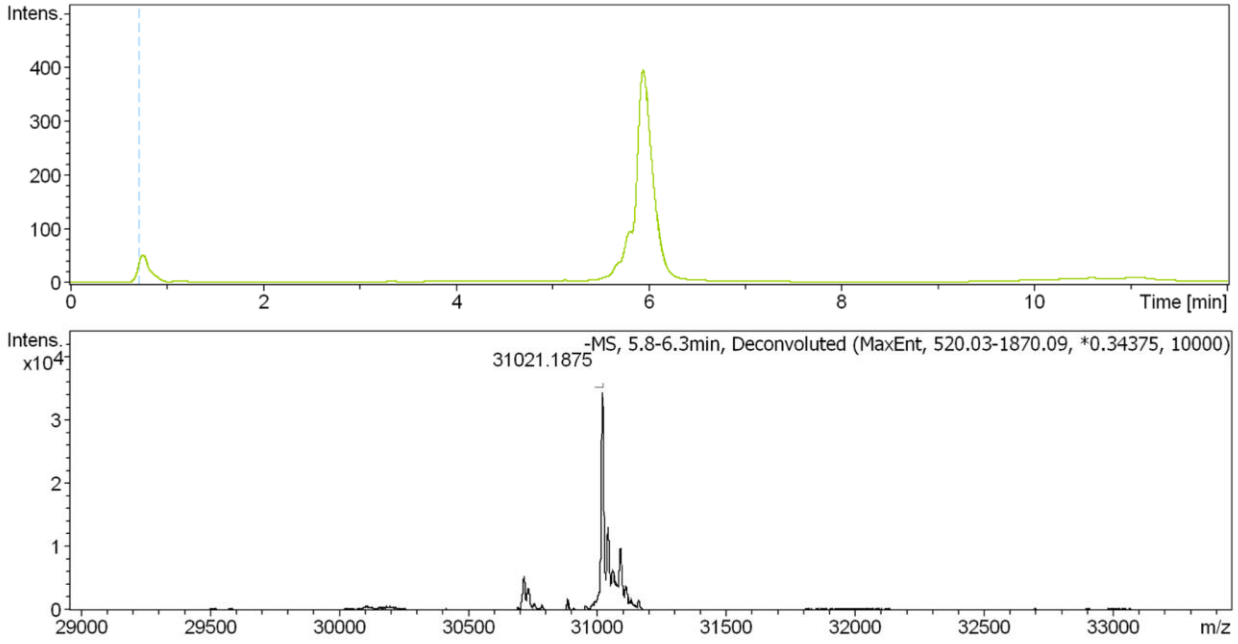

Figure S1 ctd. RP-HPLC traces of modified oligonucleotide strands (UV detection, 260 nm) and MS characterization.

FM-Cy3

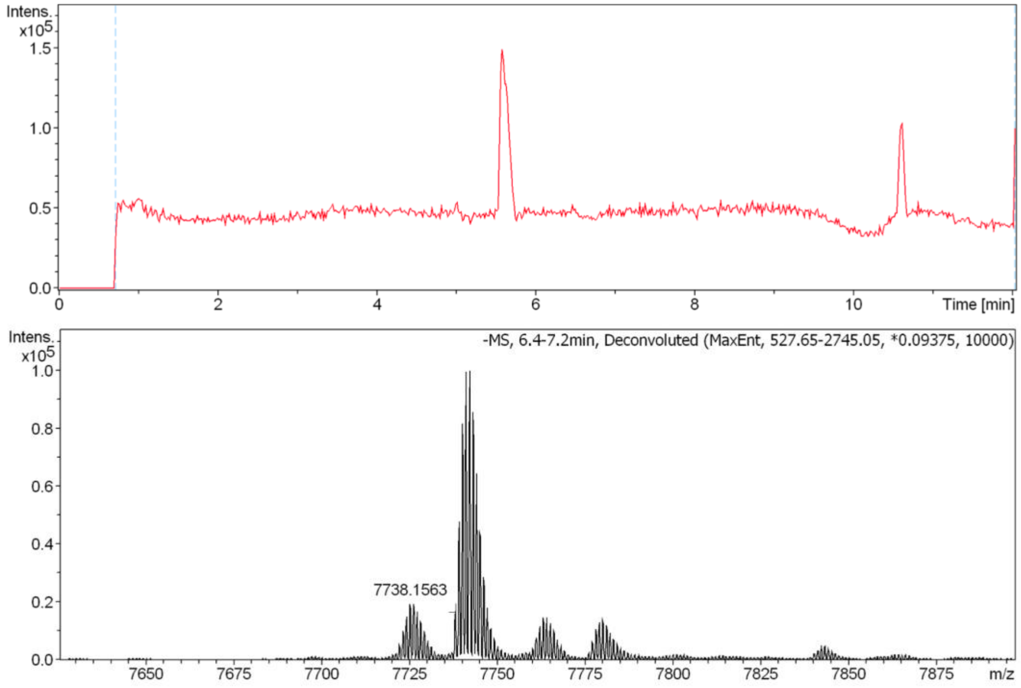

Figure S1 ctd. RP-HPLC traces of modified oligonucleotide strands (UV detection, 260 nm) and MS characterization.

8. Gel Electrophoresis Analysis

<u>Cube Assembly Characterization</u>

6% native polyacrylamide gel electrophoresis (PAGE) was carried out at 4 ºC for 90 min at 80 V in 1x TAMg buffer. For each lane 2 µL (1 µM) of sample in water was added to 1 µL of glycerol in water. Samples that were doped with Cy3 strands were first imaged on the Cy3 channel, followed by staining with GelRed® and imaging on the GelRed® channel.

2% agarose gel electrophoresis was carried out at 4 ºC for 2 hours at 80 V in 1x TAMg buffer. For each lane 2 µL (1 µM) of sample in water was loaded. Samples that were doped with Cy3 strands were first imaged on the Cy3 channel, followed by staining with GelRed® and imaging on the GelRed® channel.

<u>Cube Denaturing and Crosslinking Characterization</u>

6% denaturing polyacrylamide gel electrophoresis (PAGE) was carried out at room temperature for 50 min at 100 V. 1x TBE buffer was used and the concentration of urea in the gel was 8 M. For each lane 2 µL (1 µM) of sample in water was added to 2 µL of 8 M urea. Samples that were doped with 10% Cy3 strands were first imaged on the Cy3 channel, followed by staining with GelRed® and imaging on the GelRed® channel.

9. Cube Crosslinking Optimization

DNA nanocubes (1 µM, 100 µL) were prepared by mixing each clip sequence (C1, C2, C3, and C4) together in 1x TAMg so that the final concentration (1 µM) of each individual strand was the same. Samples were then annealed over 6 hours from 95 ºC to 4 ºC. The annealed sample was then split into 10 µL aliquots where 1 µL of varying concentrations of TCEP were added (0, 1.25, 2.50, 5.0, 10.0, 20.0, 100 mM) to each 10 µL aliquot nanocube in 1x TAMg. Samples were then incubated at 37 ºC for 24 and 48 hours. Crosslinking at each stage was characterized by 6% denaturing PAGE.

Complete crosslinking of the nanocube was obtained by incubating the sample with an excess of TCEP (10 mM). Samples were incubated for 1 hour to allow for full cleavage of 1-hexylthiol chains to take place. Afterwards, the excess TCEP was filtered out using amicon filtration with a 3K Dalton filter. Successive filtrations were performed in 1x TAMg to allow for complete removal of reducing agent and subsequent reoxidation to take place.

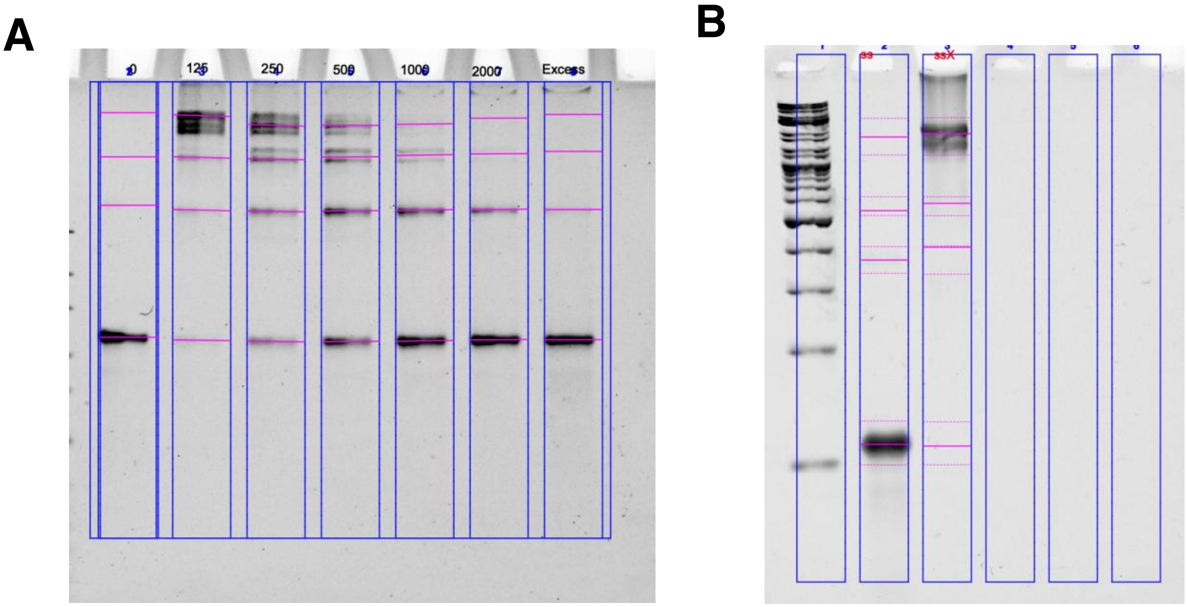

Figure S2. Quantification of crosslinking yields via band intensity in 6% denaturing PAGE gels after A) 48 hour incubation with varying concentrations of TCEP and B) incubation with an excess of TCEP followed by amicon filtration.

10. Dynamic Light Scattering (DLS)

DNA Nanocubes (20 µL, 2 µM) were prepared in 1x TAMg buffer. The solutions were analyzed by dynamic light scattering on a Malvern Panalytical Zetasizer Nano ZS.

Table S2. Average size of Cubes based on DLS analysis. *Presence of large aggregates increase the PDI.

	Average size (nm)	PDI
Non-crosslinked	9.92 ± 0.92	0.481*
Crosslinked	17.38 ± 4.18	0.513*

11. Thermal Denaturation

For thermal denaturation experiments, 100 µL of either non-crosslinked or crosslinked cubes were prepared at 0.5 µM. Samples were injected into a cuvette and placed in a Cary 3500 UV vis which sequentially tracked the absorbance of DNA at 260 nm upon heating from 25 to 95 at a rate of 1 ºC/min. Melting temperatures of each structure were determined by the machine based on the temperatures of the maximal first derivative. For cooling experiments, samples were heated from 25 to 85 ºC at a rate of 1 ºC/min followed by a cooling cycle from 85 to 25 ºC.

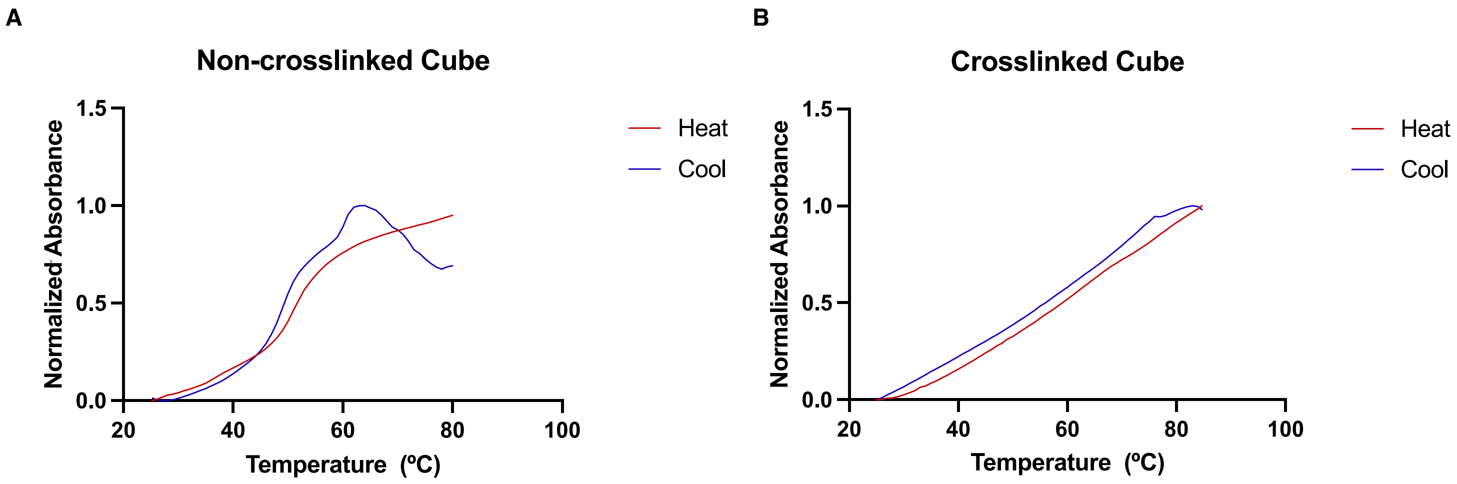

Figure S3. A) Thermal denaturation of A) non-crosslinked and B) crosslinked nanocubes by measuring absorbance as temperature was increased from 25 ºC to 85 ºC for heating (red) followed by cooling (blue) from 85 ºC to 25 ºC.

Samples of non-crosslinked and crosslinked cube were also prepared at 2 µM, then either maintained at room temperature or heating at 85 ºC for 15 min. Afterwards, both room temperature and heated samples were analyzed on a 6% native PAGE in 1x TAMg that ran at 80V for 1h.

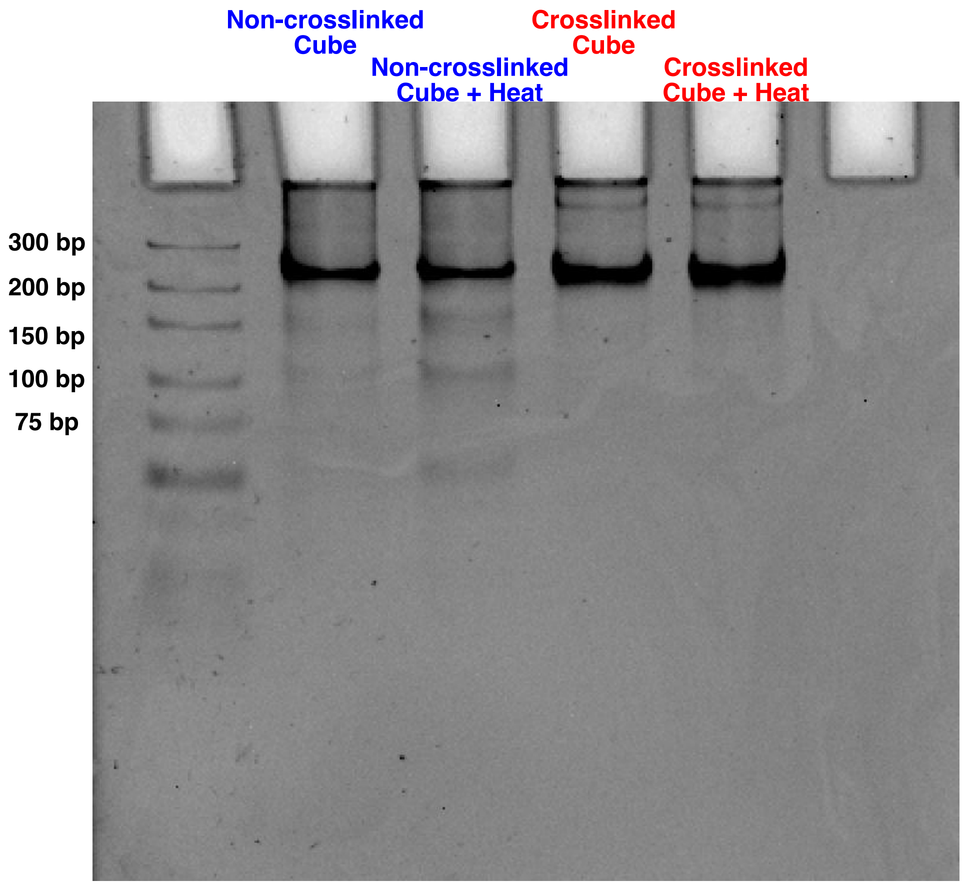

Figure S4. 6% Native PAGE in 1x TAMg of non-crosslinked and crosslinked cubes maintained at room temperature (lanes 1 and 3 respectively) or non-crosslinked and crosslinked cubes heated for 15 minutes at 85 ºC (lanes 2 and 4 respectively).

12. Electrophoretic Mobility Shift Assay (EMSA)

For protein binding EMSA, samples were first assembled by annealing over 6 h from 95 °C to 4 °C. 1 µL (2 µM) of each sample was then diluted to 10 µL with either 1x TAMg, human serum albumin (HSA), or DMEM containing 10% FBS supplemented with antibiotic/antimycotic. Samples were allowed to incubate for 1 hour. For the albumin samples a stock 75 µM solution of HSA was prepared and 9 µL of this solution was added to each 1 µL cube sample (2 µM). 10 µL samples were then loaded on a 2% agarose gel in 1x TAMg.

13. Size Exclusion Chromatography (SEC)

Samples were prepared according to the aforementioned crosslinking procedure at a final concentration of 2 µM. For samples of non-crosslinked and crosslinked nanocubes that were not incubated with HSA or FBS, 5 µL of 1 µM solutions in 1x TAMg were injected. Samples incubated with HSA (75 µM) or 10% FBS were prepared at 2.5 µL (2 µM), then diluted in either 2.5 µL of 75 µM HSA or 10% FBS in DMEM before being incubated for 1 h at 37 °C and injected on an Agilent AdvanceBio SEC column (2.7 µm, 300 mm, 300 Å, 1x TAMg buffer, flow rate 0.3 mL/min, 20 min run).

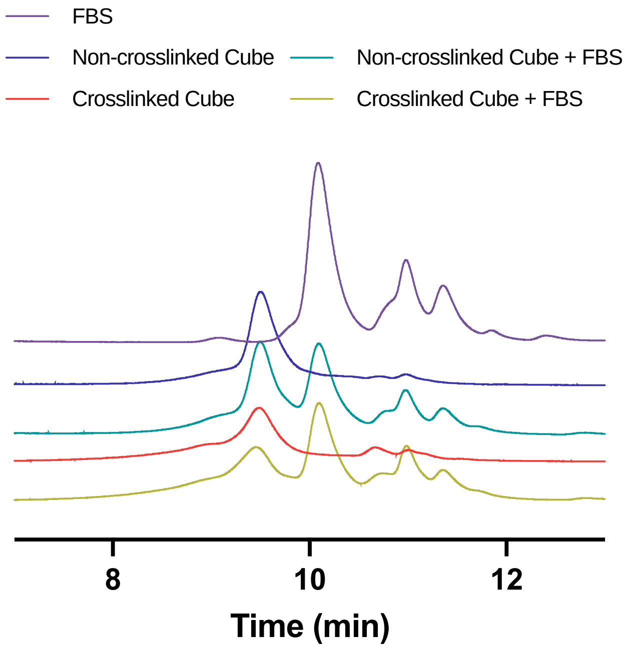

Figure S5. Size exclusion chromatogram in 1x TAMg displaying retention times of 10% FBS in DMEM and the non-crosslinked and crosslinked cube, with and without preincubation in 10% FBS in DMEM.

14. Nuclease Cleavage Assays (DNAseI, ExoI, ExoIII)

Samples were prepared containing 1µL of nanocube at 1 µM in 1x DNAseI, ExoI or ExoIII Buffer. Varying enzyme concentrations were used to optimize conditions. Samples were then incubated at 37 ºC and aliquots were taking at each time point. Enzyme activity was

terminated at each time point by freezing at -20 ºC. After taking all time points, enzyme was degraded upon incubation at 37 ºC for 2 h using 1 µL of 20 mg/µL of Proteinase K. Samples were then analyzed by 6% native PAGE (80 V, 90 min). After determining optimal conditions, the same assay was repeated on both non-crosslinked and crosslinked nanocubes.

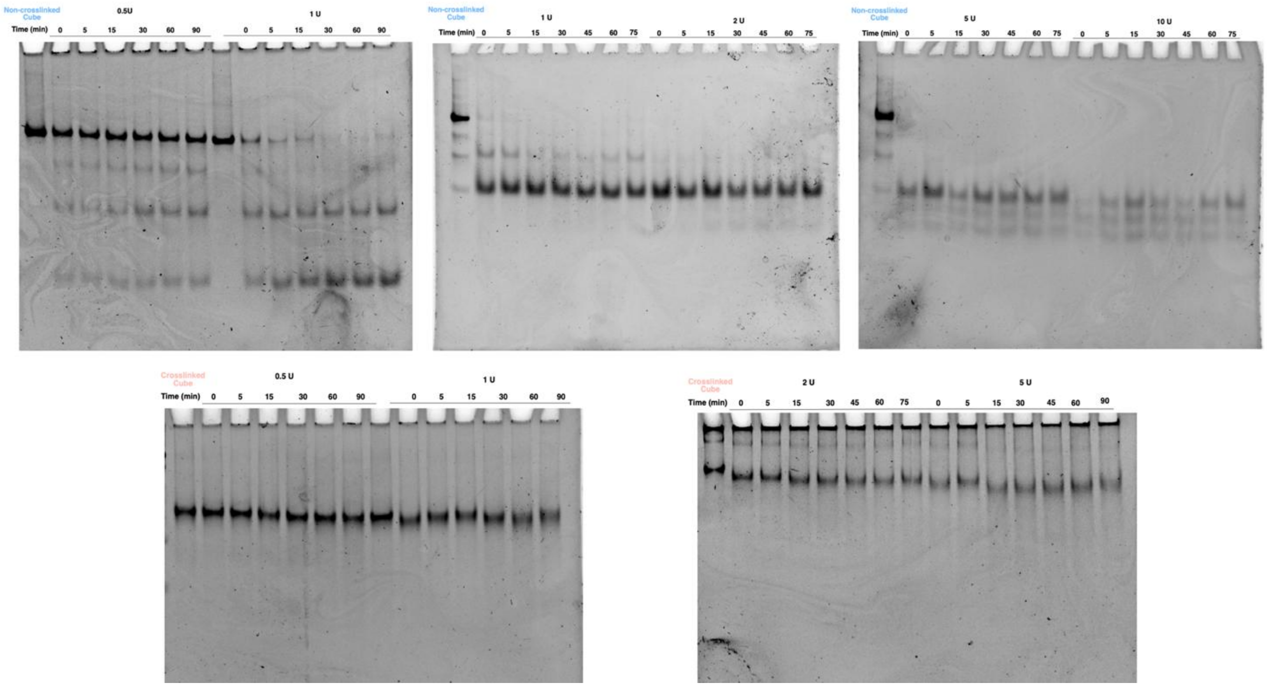

Figure S6. Incubation of non-crosslinked (blue), and crosslinked cubes (pink) for different times with varying concentrations of exonuclease III (0.5, 1, 2, 5 and 10 U) for non-crosslinked cube and (0.5, 1, 2, and 5 U) for crosslinked cube. Samples were run on a 6% Native PAGE gel in 1x TAMg run at 80 V for 90 mins.

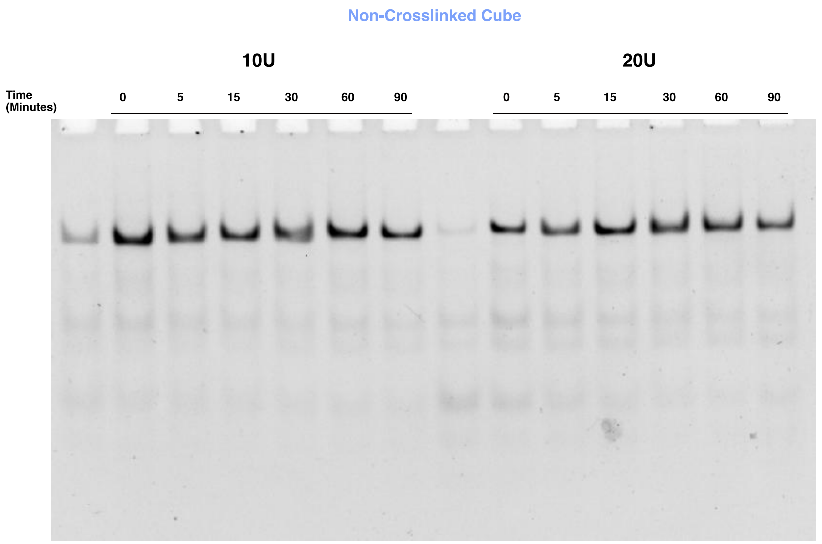

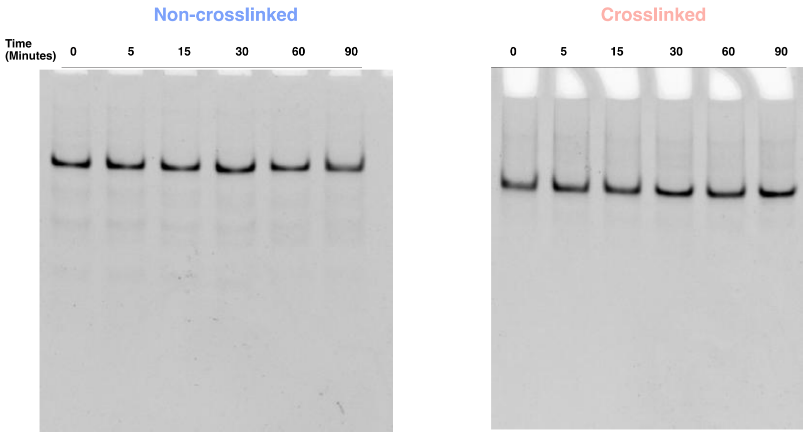

Figure S7. A) Incubation of non-crosslinked (blue), with varying concentrations of exonuclease I (10 and 20 U) for varying amounts of time. B) Incubation of non-crosslinked cube (blue) and crosslinked cube (pink) with an enzyme concentration of 10 U for varying amounts of time. Samples were run on a 6% Native PAGE gel in 1x TAMg run at 80 V for 90 mins.

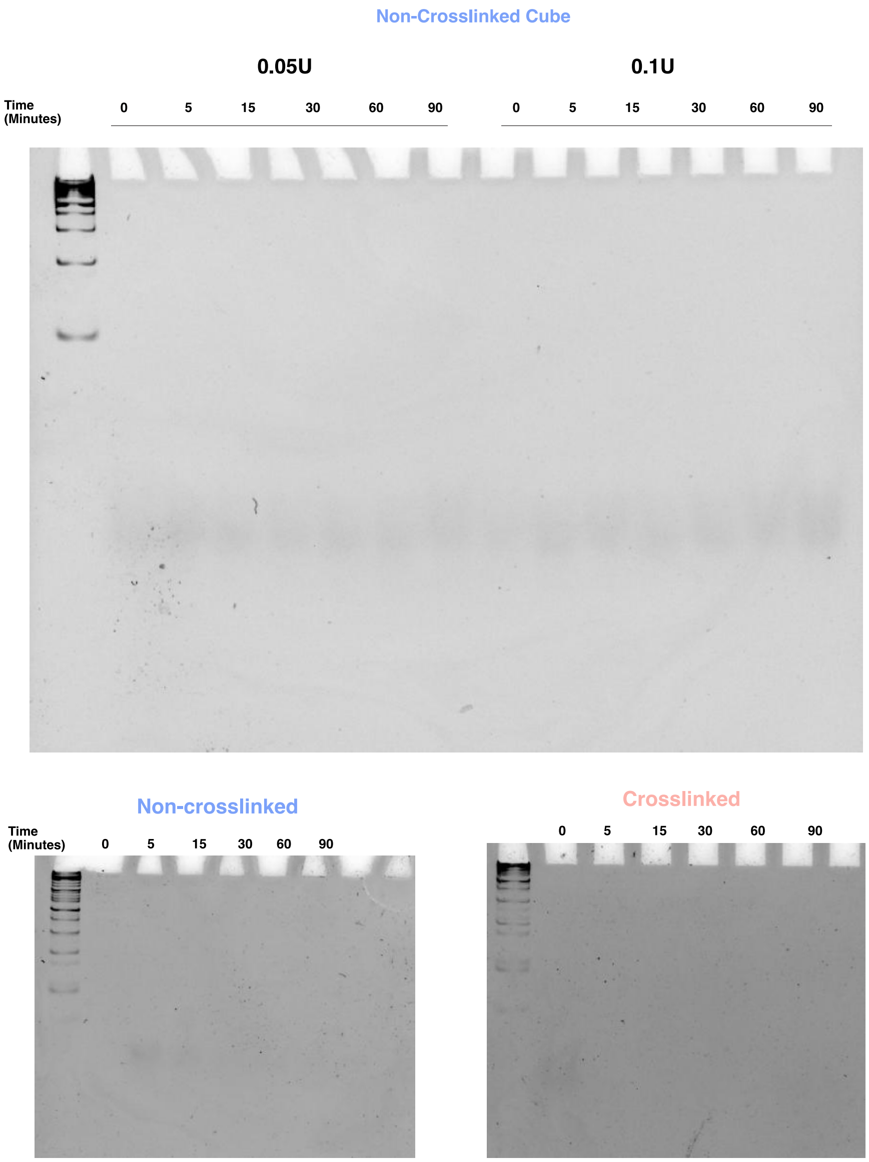

Figure S8. A) Incubation of non-crosslinked (blue), with varying concentrations of DNAse I (0.05 and 0.1 U) for varying amounts of time. B) Incubation of non-crosslinked cube (blue) and crosslinked cube (pink) with an enzyme concentration of 0.05 U for varying amounts of time. Samples were run on a 6% Native PAGE gel in 1x TAMg run at 80 V for 90 mins.

15. Serum Stability

Non-crosslinked and crosslinked cubes were prepared at 2 µM according to the aforementioned procedure. Samples were then diluted with 10% FBS DMEM to achieve a final concentration of 200 nM. 10 µL aliquots were then incubated at 37 ºC for 0, 1, 2, 4, 6, 8, 12 and 24 h and frozen at -20 ºC to quench enzyme activity at each time point. After completion, samples were treated with 1 µL of 20 mg/mL proteinase K enzyme at 37 ºC for 2 h in order to degrade the enzymes in serum. 10 µL aliquots were then loaded with glycerol on a 6% Native PAGE gel in 1x TAMg run at 80V for 90 mins at 4 ºC. Changes in band intensity of nanostructure with time were tracked by gel.

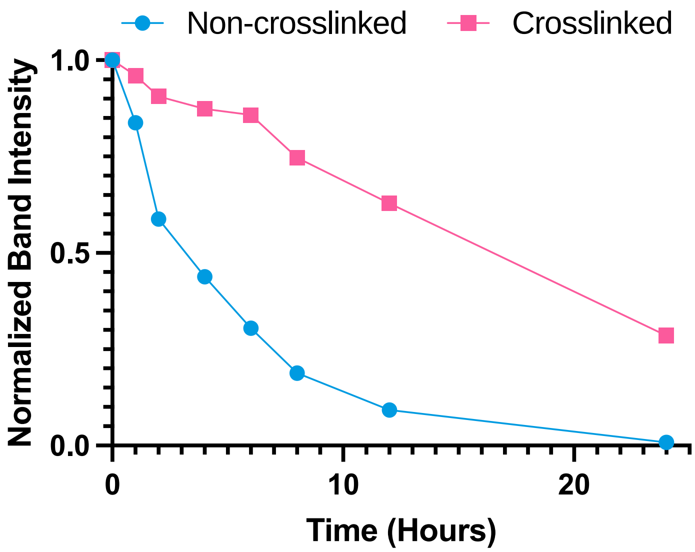

Figure S9. Quantification of changes in band intensity with time found in serum stability gels for non-crosslinked cube (blue) and crosslinked gels (pink) found in Figure 5 in manuscript.

16. Flow Cytometry and Uptake Inhibition

HeLa cells were seeded at a density of 50,000 cells in a 24-well plate. After 24 h, the cells were incubated (without transfection) with the corresponding samples that were doped with 10% Cy3-labeled strands at a final concentration of 1 µM for varying time points (1, 3 and 6 h). Then, cells were detached, washed, and resuspended in 1x PBS and processed using a BD Accuri^{TM} C6 Plus Flow Cytometer. All measurements were performed in duplicates.

Flow-cytometry gating was done to isolate single cells, separating cell debris and doublets. Gating was first done by selected to exclude cell debris looking for cells with a consistent shape by comparing front scatter area and side scattered area.

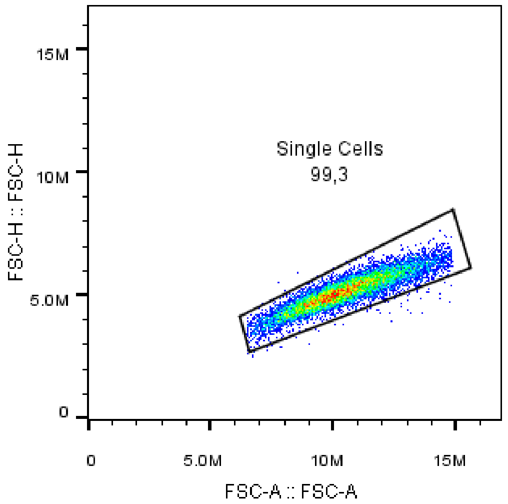

To select for only single cells and eliminate doublets front scatter area was compared to front scatter height. Cells along the diagonal were selected for due to the linear relationship

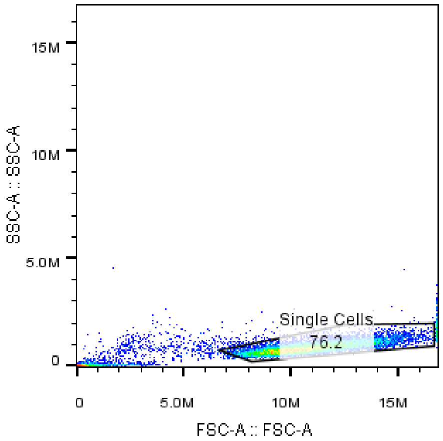

between height and area.

Following gating mean fluorescence intensity was measured.

For uptake inhibition experiments, cells were incubated with the specific uptake inhibitor 30 minutes prior to incubation with the sample. Final concentrations of inhibitor used were the following: polyinosinic acid (PI) (500 μg/mL), sucrose (15 mM), methyl-β-cyclodextrin (mBCD) (3 mM), 5,5′-dithiobis-(2-nitrobenzoic acid) (DTNB) (1.2 mM), and cytochalasin D

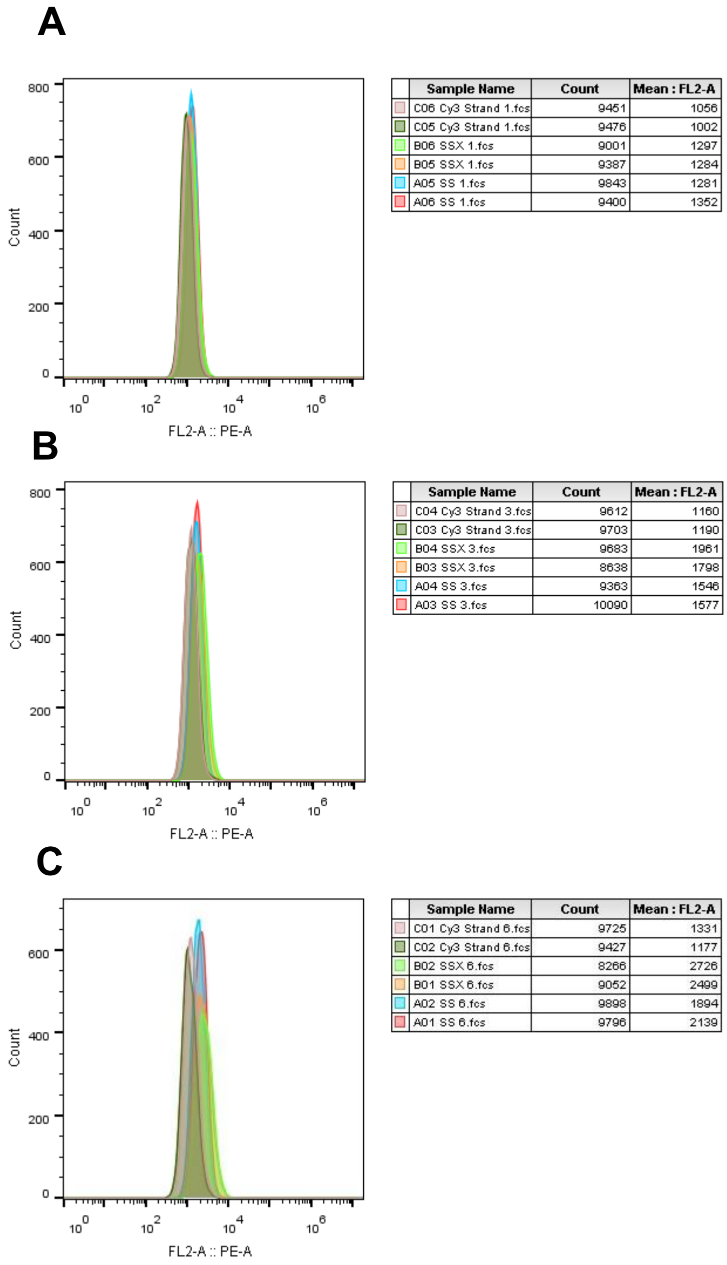

(CytoD) (10 μM).

Figure S10. Mean fluorescence intensities as a function of cell count for non-crosslinked cube (SS), crosslinked cube (SSX) and Cy3 Probe at A) 1 hour, B) 3 hour, and C) 6 hour incubation with corresponding sample.

A

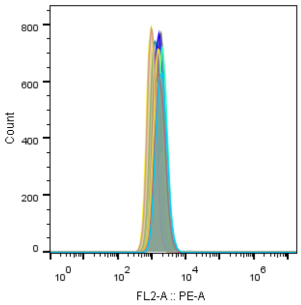

	Sample Name	Count	Mean : FL2-A
	B04 SSX 3.fcs	9683	1961
	B03 SSX 3.fcs	8638	1798
	A05 SSX PI.fcs	9532	1002
	A06 SSX PI.fcs	9549	959
	B05 SSX Sucrose.fcs	9318	1792
	B06 SSX Sucrose.fcs	8905	1661
	D05 SSX CytoD.fcs	4576	2237
	D06 SSX CytoD.fcs	9011	2190
	A04 SS 3.fcs	9363	1546
	A03 SS 3.fcs	10090	1577
	A01 SS PI.fcs	9685	1244
	A02 SS PI.fcs	8514	1395
	B01 SS Sucrose.fcs	8821	1631
	B02 SS Sucrose.fcs	7881	1636
	D01 SS CytoD.fcs	9605	1799
	D02 SS CytoD.fcs	7872	1785

B

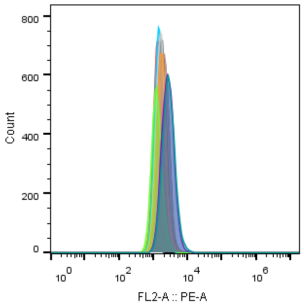

	Sample Name	Count	Mean : FL2-A
	B04 SSX 3.fcs	9612	2978
	B03 SSX 3.fcs	9875	2777
	A04 SSX M.fcs	7300	1302
	A03 SSX M.fcs	8424	1782
	D06 SSX DTNB.fcs	9243	1668
	D05 SSX DTNB.fcs	9150	1749
	A03 SS 3.fcs	9490	2085
	A04 SS 3.fcs	9426	2174
	A02 SS M.fcs	9048	1478
	A01 SS M.fcs	9013	1419
	D04 SS DTNB.fcs	406	1036
	D03 SS DTNB.fcs	1296	987

Figure S11. Mean fluorescence intensities as a function of cell count for non-crosslinked cube (SS) and crosslinked cube (SSX) after incubation in serum for 3 h before preincubation with **A)** polyinosinic acid (500 ng/µL), sucrose (15 mM) or cytochalasin D (10 µM) and with **B)** methyl-β-cyclodextrin (mBCD) (3 mM) or 5,5′-dithiobis-(2-nitrobenzoic acid) (DTNB) (1.2 mM).

17. Cellular Cytotoxicity

Without Transfection Agents: HeLa cells were counted and seeded at a density of 100k cells/mL in a 96-well plate and were allowed to recover for 24 h at 37º C with 5% CO_2. Non-crosslinked and crosslinked nanocubes were prepared at a concentration of 1.25 µM, and were then added to the appropriate well in six replicates to achieve a final concentration of 125 nM of nanocube in each well. Cells were incubated for a total of 24 h (post-DNA addition).

With Transfection Agents: HeLa cells were counted and seeded at a density of 100k cells/mL in a 96-well plate and were allowed to recover for 24 h at 37º C with 5% CO_2. Non-crosslinked and crosslinked nanocubes were prepared at a concentration of 1.25 µM, where Lipofectamine™ 3000 reagent was used as transfection agent and control (Invitrogen) following the vendor's procedure (using Optimem as transfection media mix) and were then added to the

appropriate well in six replicates to achieve a final concentration of 62.5 nM. Cells were incubated for a total of 24 h (post-DNA addition).

For cytotoxicity and cell viability, cells were incubated with 25 µL of Cell-Titer Blue for 1.5 h in 5% CO_2 at 37° C after 24 h of incubation with samples. Subsequently, 96-well plates were allowed to equilibrate at room temperature and the fluorescence was measured at 590 nm (Ex. 530, Em. 590) using a BioTek Synergy HT microplate reader. All quantifications were done using GraphPad Prism 9 software.

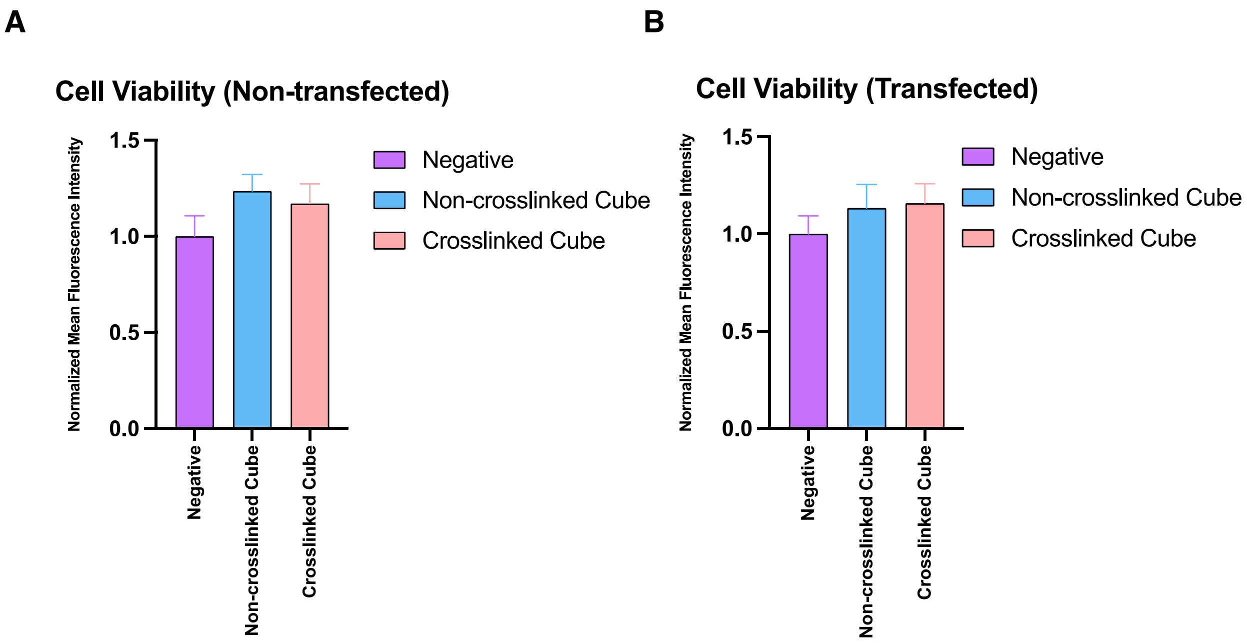

Figure S12. Fluorescent measurements of Cell Titer-Blue corresponding to the viability of cells incubated with non-crosslinked cube (blue) or crosslinked cube (pink), normalized to the negative control of 1x TAMg buffer. This was done using either a final concentration of nanocube of 125 nM without any transfection agents (A) or at a final concentration of nanocube of 62.5 nM, but with the use of transfection agents (B).

17. Supporting References

[1] Faiad, S.; Laurent, Q.; Prinzen, A. L.; Asohan, J.; Saliba, D.; Toader, V.; Sleiman, H. F. Impact of the Core Chemistry of Self-Assembled Spherical Nucleic Acids on Their In Vitro Fate. *Angewandte Chemie* **2023**, *135* (51), e202315768
